# The Preference-Expectation Gap in Support for Female Candidates: Evidence from Japan

**DOI:** 10.1093/poq/nfaf002

**Published:** 2025-05-23

**Authors:** Gento Kato, Fan Lu, Masahisa Endo

**Affiliations:** Senior Assistant Professor, School of Political Science and Economics, Meiji University, Tokyo, Japan; Assistant Professor, Department of Political Studies, Queen’s University, Kingston, ON, Canada; Professor, Faculty of Social Sciences, Waseda University, Tokyo, Japan

## Abstract

Gender disparities in Japanese government are consistently high, but evidence of voter bias against female politicians is mixed. We argue that this discrepancy arises because some researchers measure Japanese voters’ first-order preferences (who they personally support) while other researchers measure Japanese voters’ second-order preferences (who they expect other voters to support). We call this gap between voters’ *own preferences* and *expectations* regarding *others’* preferences the preference-expectation gap. Since this gap is a key mechanism of strategic discrimination, we test our argument using an experimental design modelled after research on strategic discrimination in the 2020 US Democratic primary elections. Based on two online conjoint survey experiments in Japan, our findings demonstrate the presence of a preference-expectation gap in Japanese public opinion on female politicians. Exploratory analyses of moderation effects reveal that female participants and those with more liberal views toward gender roles have larger preference-expectation gaps.

Public opinion on gender representation in American government consistently expresses a preference for female over male candidates (Dolan 2014a, 2014b; Hayes and Lawless 2016). This preference holds true outside the United States as well (Schwarz and Coppock 2022). Yet, women remain underrepresented in governments across the world (Hughes 2013; Inter-Parliamentary Union 2023).

Since there does not seem to be a lack of “demand” for female politicians, researchers have sought explanations for their underrepresentation in the “supply” of female candidates (Teele, Kalla, and Rosenbluth 2018; Piscopo 2019; Bernhard, Shames, and Teele 2021; Phillips 2021). However, discussions of “strategic discrimination” in the 2020 US Democratic presidential primaries suggest it may be incorrect to equate voter “demand” with their preferences (Bateson 2020; Green, Schaffner, and Luks 2023). Even though Democratic voters personally preferred female over male candidates, they favored male over female candidates in terms of their potential to beat Donald Trump in the general election. This pattern implies that voters’ preferences need not match their demand/vote choice if there is a gap between their *own preferences* and their *expectations* regarding *other* voters’ preferences. We call this gap the preference-expectation gap.

This research note assesses the preference-expectation gap in a vastly different political and cultural context: evaluations of female politicians in Japan. Evidence is mixed on whether Japanese voters are willing to choose female politicians. Kawato (2007) and Ono and Yamada (2020) find systematic bias against them, while Kage, Rosenbluth, and Tanaka (2019) and Horiuchi, Smith, and Yamamoto (2020) do not. If there is a preference-expectation gap in how voters evaluate female politicians, both sets of evidence can hold true. As Bateson (2020) and Green, Schaffner, and Luks (2023) show, expectations about other people’s objections to the candidate’s identity can influence voter decisions. Even when voters *prefer* to have women in politics, they may not *support* women if they expect women to be unpopular among other voters. Consistent with this idea, among existing empirical studies of Japanese voters, those finding bias against women tend to ask voters which candidate they would *support*, whereas those finding no bias against women tend to ask which candidate they would *prefer.*^1^ Based on the discussion above, we form the following hypotheses:^2^

*H1. Japanese voters personally prefer female candidates no less than male candidates.*

*H2. Japanese voters expect other voters to prefer female candidates less than male candidates. In other words, they perceive female candidates as less electable (i.e., less likely to win elections) than male candidates.*

The extension of the preference-expectation gap concept to Japan is important for at least three reasons. First, Japan has much worse gender imbalances in government than in the United States. In 2023, the World Economic Forum ranked Japan 138 out of 146 countries with regard to gender parity in politics and 125 on gender parity overall. The United States ranks 63 and 43 on these same rankings (World Economic Forum 2023). Therefore, studying gender bias is an even more pressing task for Japanese researchers and practitioners. Second, there is a strong culture of compromising personal preferences in favor of societal expectations in Japan, which can exacerbate any detrimental consequences of the preference-expectation gap. For example, Hashimoto and Yamagishi (2015) compare the preference-expectation gap in evaluations of interdependent persons among Japanese and American subjects. They find that Japanese subjects exhibit larger preference-expectation gaps than American subjects, and that Japanese subjects’ evaluations are more strongly influenced by their expectations of others than American subjects’ evaluations. So far, such mentalities are reflected in social behavior such as test evaluations (Yamagishi, Hashimoto, and Schug 2008; Hashimoto, Li, and Yamagishi 2011; Yamagishi et al. 2012), but we expect they affect political behavior as well. Third, the formation of expectations in multiparty parliamentary elections in Japan provides a better context for isolating gender stereotypes as a causal mechanism. In the 2020 US Democratic primaries, it was relatively straightforward for voters to presume that female candidates have lower chances of beating male candidates in the general election, because they knew with certainty the general election involved Donald Trump and Republican voters. In Japan, there are no clear heuristics such as “Republican” and “Trump.” Therefore, we believe that the preference-expectation gap in Japan, if it exists, is more attributable to genuine gender stereotypes among voters.

## Study Design

We implement two conjoint experiments modeled after Green, Schaffner, and Luks (2023) to test our proposed hypotheses. We recruited participants through the online survey company *Rakuten Insight* and collected responses using *Qualtrics. Rakuten Insight* provided us with a nonprobability sample of participants (all at least 18 years old) who entered their survey pool through advertisement on affiliate websites, banners, referrals, and social media platforms. The sampling balances the joint distribution of gender, age, and region, to match statistics from the Japanese census. Cooperation rates (number of completed questionnaires divided by the number of contacts with eligible individuals; AAPOR COOP1) are 9.7 percent and 10.0 percent for Experiments 1 and 2, respectively. All analyses are unweighted.

Both conjoint experiments comprise tasks that require participants to choose one of two randomly generated hypothetical profiles of political candidates. The profiles include gender, party affiliation, age, political experience, education level, marital status, number of children, and residence in proximity to parents. The last two attributes—whether political candidates have children and whether they live with parents—are relevant considerations in an aging society such as Japan (see Supplementary Material section A for more details). Each participant completes six to eight tasks. The first three to four iterations of the task measure *preferences*: participants choose the profile that is “more desirable” (望ましい). The next three to four iterations measure *expectations*: participants choose the profile that is “more likely to win” (勝利しそう).^3^ To avoid order effects, we reverse the order of task types for a random half of the participants.

We fielded the first experiment between January 21 and 25, 2022. This experiment recruited 1,803 participants who each completed six tasks (three for each task type). After excluding missing responses, we have 10,606 cases for “preference tasks” and 10,612 cases for “expectation tasks.”^4^ We fielded the second experiment between March 16 and 24, 2022. This experiment recruited 2,406 participants who each completed eight tasks (four for each task type). It builds on results from the first experiment in two key ways.^5^ First, our second experiment includes policy focus as a randomly assigned candidate attribute. Policy focus can be a confounding factor because “compassion” issues such as education and poverty are stereotypically associated with female competency while foreign policy and military defense are associated with male competency (Huddy and Terkildsen 1993; Swers 2007). Second, while our first experiment is situated within the context of the Japanese House of Representatives, half of the participants in the second experiment select profiles of candidates competing in a hypothetical municipal council election. Here, voters may make different trade-offs between their preference and expectation in local rather than national elections. Local elections in Japan are expected to be more personalized than national elections, with a majority of candidates running as independents in multi-member districts. Also, gender stereotypes may advantage Japanese women in local elections (Eto 2005; Nakano 2018).^6^ After excluding missing responses, we have 9,558 cases for “preference tasks” and 9,492 cases for “expectation tasks” in the hypothetical House of Representatives election; we have 9,420 cases for “preference tasks” and 9,358 cases for “expectation tasks” in the hypothetical municipal council election.

## Findings

**Figure 1.**
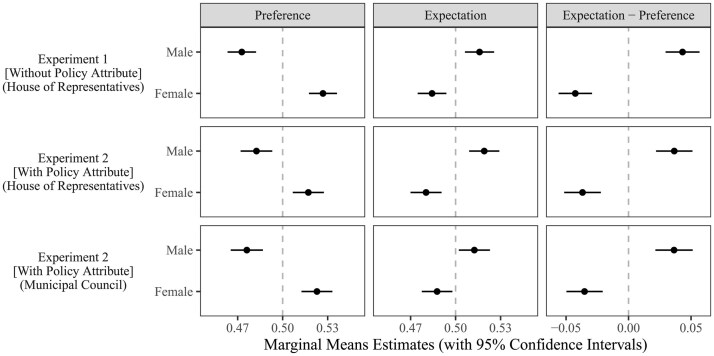
Individuals prefer female political candidates more than male ones, but they expect females to have lower chances to win elections than males.

Figure 1 presents main results regarding the effect of candidate gender (see Supplementary Material section B for full results). To assess H1 and H2, we calculate marginal means, which indicate the probability participants select profiles with a certain attribute level, ignoring all other attributes. All estimations are made with cluster-robust standard errors using the cj function in the cregg package of the statistical software R (Leeper, Hobolt, and Tilley 2020). The left column presents marginal means for “preference tasks.” In support of H1, participants prefer female over male profiles on average across all experimental conditions. Magnitudes of coefficient estimates are very similar across conditions, ranging between 3.5 to 5.4 percentage points. This result indicates preference for female candidates holds regardless of gendered policy competence stereotypes.

The center column of figure 1 focuses on choices in “expectation tasks.” In support of H2, the pattern flips completely from “preference tasks.” In Experiment 1, we see a 3.1 percentage point advantage of male profiles over female profiles in expected chances of winning the House of Representatives election. In the first condition of Experiment 2, such a female disadvantage remains intact even after controlling for the confounding effects of policy focus. The female disadvantage is 1.4 percentage points smaller in the second condition of Experiment 2, but there remains an expectation that male candidates are significantly more likely to win than female candidates in a municipal council election.

To quantify the size of the preference-expectation gap, the right column of figure 1 subtracts the marginal means in preference tasks from the marginal means in expectation tasks. A positive value indicates the expectation for a candidate’s electoral victory is higher than the preference for them to win; a negative value indicates the reverse.^7^ In Experiment 1, on average, male profiles are 4.3 percentage points more likely to be chosen under “expectation tasks” than “preference tasks”; female profiles are 4.3 percentage points less likely to be chosen under “expectation tasks” than “preference tasks.” In both conditions of Experiment 2, preference-expectation gaps slightly shrink but stay substantial at 3.5 to 3.7 percentage points on average. These gaps are all highly statistically significant at *p < *0.001.

## Exploring Moderation Effects

To deepen our understanding of mechanisms driving the preference-expectation gap in support for female politicians in Japan, we explore whether the magnitude of this gap differs by participants’ own attributes as well as candidates’ partisanship.^8^ First, we assess the moderating role of participants’ attitudes toward gender roles. Green, Schaffner, and Luks (2023) find that differences in the preference-expectation gap for female and male candidates are largest among American voters who score low on sexism. Since sexism is more prevalent in Japan, we conduct analyses to see if the preference-expectation gap for female candidates is widest among Japanese participants who have relatively liberal views on gender roles. We rely on a battery of nine questions that tap into traditional versus liberal attitudes regarding marriage and family, asked prior to the experimental tasks (see Supplementary Material section D.1 for Japanese question wordings).^9^ Participants who hold traditional (liberal) attitudes tend to agree (disagree) with the view that marriage is preferred and, if married, the male spouse should work and earn money while the female spouse should stay home and raise children. We create a binary variable of traditional versus liberal gender role attitudes by splitting the factor analysis-based gender role attitudes score at the median.^10^

**Figure 2.**
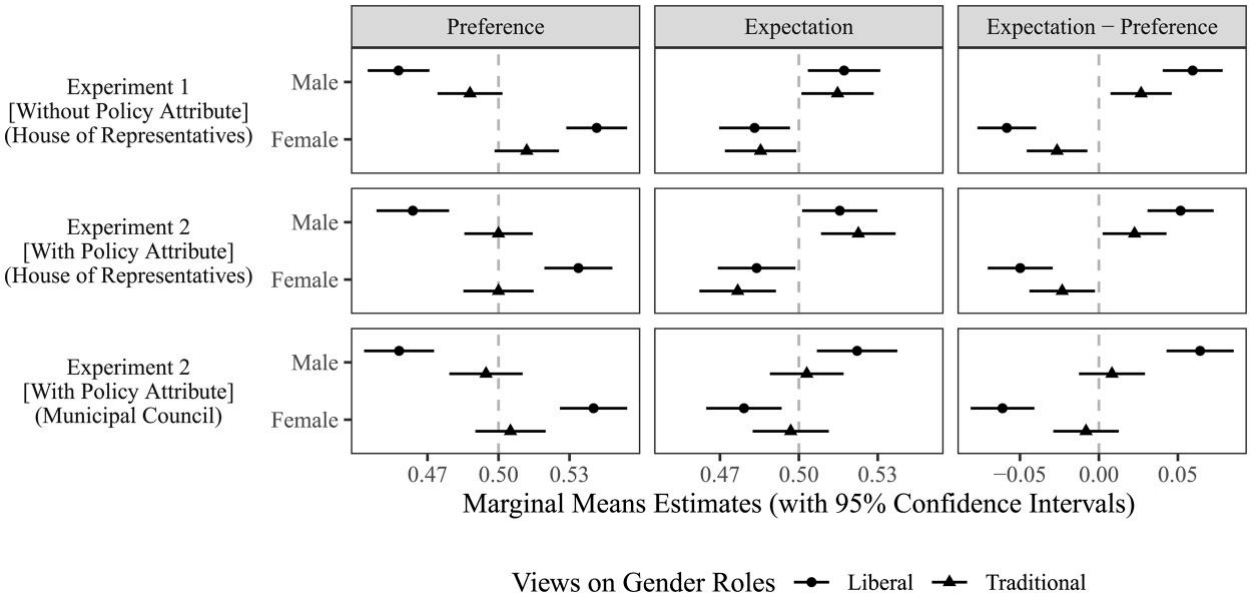
Preference-expectation gap in support for female candidates is larger for those with a liberal gender role view, because they prefer women more strongly but expect women to be no more advantaged compared to those with a traditional gender role view.

Figure 2 replicates the main results in figure 1 except that we apply the analyses to subsets of participants with traditional versus liberal views on gender roles. The left column shows that participants with traditional gender role attitudes prefer female candidates less and male candidates more than those with liberal gender role attitudes. In contrast, the center column shows virtually no difference across gender role attitudes regarding expectations of female versus male candidates’ electoral performance, especially in the national election (top two panels). Regardless of their views toward gender roles, participants believe that male candidates perform better than female candidates. In the municipal election (center column, bottom panel), we do see differences in expectations by gender role attitudes. However, it is participants with traditional gender role views who hold less gender-stereotyped expectations, while liberal participants’ expectations remain gender stereotyped. The right column illustrates that in both national and municipal elections, the preference-expectation gap is widest among Japanese participants with liberal views toward gender roles. Participants with traditional views exhibit a relatively small preference-expectation gap, and at the municipal election level, such a gap is almost zero. Our findings replicate the implication from figure 2 of Green, Schaffner, and Luks (2023, p. 892).

Next, we assess the moderating role of participant’s own gender. Similar to those with liberal attitudes toward gender roles, female voters may exhibit a wider preference-expectation gap for female candidates than male voters. Supplementary Material section E presents the results of our analyses. We find it is indeed female participants who are driving the preference-expectation gap across all experimental conditions. Female participants prefer female candidates significantly more than male participants, but they are much more likely to expect other participants to prefer male candidates.^11^

Finally, we assess the moderating role of candidate’s party affiliation. Since the proportion of women in ruling parties such as the Liberal Democratic Party is much lower than that of opposition parties (The Asahi Shimbun 2019), partisanship may be a heuristic for candidate quality, which in turn moderates the magnitude of the preference-expectation gap. Supplementary Material section F presents the results of our analyses. We find that participants expect ruling party candidates to perform better than opposition party candidates, but they do not necessarily prefer ruling party candidates. As a result, we see a preference-expectation gap with respect to partisanship as well. Setting aside the direct effect of partisanship, differences in the magnitude of the preference-expectation gap based on candidate’s gender persists regardless of affiliated party. In other words, we observe no systematic moderation effect of candidate’s party on the preference-expectation gap in support for female candidates.

## Discussion

Building upon research on strategic discrimination in the United States (Bateson 2020), we examine if there is a disconnect between Japanese voters’ “first and second order preferences” (Green, Schaffner, and Luks 2023, p. 887). Evidence from two online conjoint experiments confirms the existence of a preference-expectation gap in support for female political candidates in Japan. Holding other attributes constant, we find that experiment participants on average personally prefer female over male politicians, but they expect females to be less likely to win elections than males. This preference-expectation gap persists across electoral contexts, alternative sets of candidate attributes, and with or without controlling for candidates’ policy focus. We also find no evidence that candidate’s party affiliation plays a moderating role. However, the magnitude of the gap varies with participants’ individual attributes. We find the preference-expectation gap is wider among female participants, and wider among participants who hold more liberal views on gender roles (in which the difference is exacerbated in local elections).

Our findings from Japan add four insights to the theory of strategic discrimination. First, the preference-expectation gap extends beyond the American context. Even in a country ranked 138 out of 146 countries in terms of gender disparities in political empowerment (World Economic Forum 2023), citizens no longer have overt biases against female politicians. At the same time, however, they appear to not have updated their beliefs on how Japanese society as a whole perceives female politicians. Second, evidence of the preference-expectation gap in the Japanese parliamentary election setting emphasizes the role of psychological biases in the formation of expectations. Third, evidence that female participants drive the gap more than male participants has important implications for the extent to which female voters could mitigate gender disparity in Japanese politics. Fourth, our finding that participants with more liberal views toward gender roles have more pessimistic expectations toward female candidates in local elections speaks to existing literature on how more educated and politically knowledgeable voters (who are potentially more liberal) tend to form stronger gender stereotypes (e.g., Koch 1999, 2002).

This research note is a first step to assessing a key mechanism of strategic discrimination in a non-American context, and there are a number of ways to move the study forward. For one, we find the preference-expectation gap in support for female politicians in Japan, but we do not yet assess how consequential it is in driving strategic discrimination against female politicians. Findings from Hashimoto and Yamagishi (2015) imply that the impact of (biased) beliefs about others can be more detrimental in Japan than in the United States, but this implication has never been tested in the context of political attitudes and behavior. It also remains untested whether preference-expectation gaps affect the political choices of women more than that of men. In addition, the type of electoral system may interact with how the preference-expectation gap affects voting. In this regard, further research is required on the behavioral consequences of the preference-expectation gap in support for women and other underrepresented minority groups in Japan, both as a single-country study and in comparison with other democracies. Finally, we believe that the logic of strategic discrimination may extend beyond voters to elites. For example, Doherty, Dowling, and Miller (2019) show how perceived electoral prospects affect candidate nomination processes in American elections. In Japan, party elites dominate most candidate nomination processes, and their decisions may also be shaped by a gap between who they personally support and who they think other party members support. Therefore, further theorizing and empirical testing are required to understand the logic and practice of strategic discrimination among Japanese political elites.

## Supplementary Material

nfaf002_Supplementary_Data

## Data Availability

Replication data and documentation are available at https://doi.org/10.7910/DVN/GJWDVM.

## Footnotes

1
Kawato (2007) and Ono and Yamada (2020) use the word 支持する (support) to ask about candidate choice in their surveys. On the other hand, Horiuchi, Smith, and Yamamoto (2020) use 衆議院議員として望ましい (preferred/desired as a Member of the House of Representatives). We cannot confirm the original Japanese wording for Kage, Rosenbluth, and Tanaka (2019), but the English translation provided by the authors is “prefer as a member of the House of Representatives.”

2 Both hypotheses are preregistered at OSF (https://osf.io/pq6tk and https://osf.io/469wn). Please refer to Supplementary Material sections G and H for details.

3 Full wording is “Which of the following two persons do you think is [more desirable as a Single Member District member/more likely to win in a Single Member District election] of the House of Representatives? Even if you are not entirely sure, please indicate which of the two you would think is [more desirable/more likely to win].” Preference task wording closely follows that of Horiuchi, Smith, and Yamamoto (2020). In Experiment 2, “House of Representatives” is replaced by “Municipal Council” for half of the participants. Also, the expectation task in Experiment 2 uses the expression 当選しそう, which is directly translated as “more likely to be elected.” See full Japanese wordings in Supplementary Material section A.

4 One participant in Experiment 1 and two participants in Experiment 2 are excluded because conjoint attributes were unassigned due to technical errors. While experiments are forced-choice without explicit nonresponse options, implicit nonresponse (skipping) is allowed following the recommendation from the institutional ethics committee. Nonresponse rates are 1.9 percent in Experiment 1, 0.6 percent in Experiment 2.

5 There are other minor differences between the two experiments. See Supplementary Material section A for details.

6 In the preregistration for Experiment 2, we also form the following hypothesis: *The preference-expectation gap is wider in national elections than in local elections.* This hypothesis is not explicitly written here since our central question in this research note is whether the preference-expectation gap exists in Japan.

7 We also conduct the analysis using Average Marginal Component Effects (see Supplementary Material section C), reaching the same conclusion.

8 There are no preregistered hypotheses for moderation effects examined in this section, and therefore the analyses are purely exploratory.

9 We use “traditional” as synonymous to “conservative” because it is more commonly used in the context of describing attitudes on gender roles in Japan.

10 We use principal factor solution with a single factor. Factor score is calculated using the Bartlett method. See Supplementary Material section D.1 for the detailed procedure.

11 The level of elections seems to matter within Experiment 2, in that male participants tend to prefer female candidates less in national elections. However, this observation is only tentative, since we also see that the preference for female candidates among male participants is weaker in Experiment 2’s national election condition than in Experiment 1.
