# Supplementary material for: The Preference-Expectation Gap in Support for Female Candidates: Evidence from Japan
Source: Public Opin Q. 2025 May 23;89(1):217–28. doi: 10.1093/poq/nfaf002 (PMC12166979; doi:10.1093/poq/nfaf002)
Supplement: nfaf002_Supplementary_Data [file nfaf002_supplementary_data.pdf]

Supplementary Materials for: The preference-expectation gap in support for female candidates: Evidence from Japan

Gento Kato (Meiji University)
Fan Lu (Queen's University)[1]
Masahisa Endo (Waseda University)

Contents

A Experimental Designs **A-2**

B Full Results of Conjoint Experiments **A-5**

C Main Results using Average Marginal Component Effect **A-11**

D Moderation by Gender Role Attitudes **A-12**
- D.1 The Set of Questions Used to Measure Gender Role Attitudes A-12
- D.2 Results using Average Marginal Component Effect A-13
- D.3 Full Results of Conjoint Experiments A-14

E Moderation by Respondent's Gender **A-20**
- E.1 Figure that is parallel to Figure 2 . . . . A-20
- E.2 Results using Average Marginal Component Effect A-21
- E.3 Full Results of Conjoint Experiments A-22

F Moderation by Candidate's Party **A-28**
- F.1 Figure that is parallel to Figure 2 . . . . A-28
- F.2 Results using Average Marginal Component Effect A-29
- F.3 Full Results of Conjoint Experiments A-30

G Pre-analysis plan for the first experiment: Preference-expectation gap in support for female candidates in Japanese parliamentary elections **A-36**

H Pre-analysis plan for the second experiment: Preference-expectation gap in support for female candidates in Japanese national and local elections **A-41**

[1] Email: fan.lu@queensu.ca.

A Experimental Designs

Experiment 1 wording (underlined texts are bolded in the survey):

- (Preference task) Which of the following two persons do you think is more desirable as a Single Member District member of the House of Representatives? Even if you are not entirely sure, please indicate which of the two you would think is desirable. (次の２人の人物のうち、どちらがより小選挙区選出の衆議院議員として望ましいと思いますか。もし、どちらが望ましいかはっきりとは言えない場合でも、どちらか一方、あえていえばより望ましいと思われる方を選んでください。) Choice: Person 1 (人物１); Person 2 (人物2)
- (Expectation task) Which of the following two persons do you think is more likely to win in a Single Member District election of the House of Representatives? Even if you are not entirely sure, please indicate which of the two you would think is winning. (次の２人の人物のうち、どちらがより衆議院議員選挙の小選挙区で勝利しそうと思いますか。もし、どちらが勝利しそうかはっきりとは言えない場合でも、どちらか一方、あえていえばより勝利しそうと思われる方を選んでください。) Choice: Person 1 (人物１); Person 2(人物2)

Table A1: Experiment 1 attributes and levels

Attribute	Levels
Gender (性別)	Male (男性); Female (女性)
Party (所属政党)	Liberal Democratic Party/LDP (自民党); Komeito (公明党);
	Constitutional Democratic Party/CDP (立憲民主党);
	Democratic Party for the People/DPFP (国民民主党);
	Japanese Communist Party/JCP (共産党);
	Japan Ishin Party (日本維新の会); Independent (無所属)
Age (年齢)	35 (35歳); 45 (45歳); 55 (55歳); 65 (65歳)
Political Experience (政治経験)	No Experience (経験なし);
	Local parliament member (地方議員);
	One term as a Diet Member (国会議員１期);
	Two terms or more as a Diet Member (国会議員２期以上)
Education (最終学歴)	High School (高校卒); College (大学卒);
	Graduate School (大学院卒)
Marital status (結婚歴)	Unmarried (未婚); Married (既婚); Divorced (離別)
Children Status (子の年齢 (一番下))	No Children (子なし); Children of 6- (６歳未満);
	Children of 6+ (６－１7歳); Children of 18+ (１８歳以上)
Residential Status (居住状況)	Live together with parent (親と共に居住);
	Live close to parent (親の近くに居住);
	Live far from parent (親から遠くに居住)

Experiment 2 wording (underlined texts are bolded in the survey):

- (Preference task, national) Which of the following two persons do you think is more desirable as a Single Member District member of the House of Representatives? Even if you are not entirely sure, please indicate which of the two you would think is desirable. (次の２人の人物のうち、どちらが小選挙区選出の衆議院 議員としてより望ましいと思いますか。もし、どちらが望ましいかはっきりとは言えない場合でも、どちらか一方、あえていえばより望ましいと思われる方を選んでください。)
  Choice: Person 1 (人物１); Person 2 (人物2)

- (Expectation task, national) Which of the following two persons do you think is more likely to be elected in a Single Member District election of the House of Representatives? Even if you are not entirely sure, please indicate which of the two you would think is likely to be elected. (次の２人の人物のうち、どちらが衆議院議員選挙の 小選挙区でより当選しそうと思いますか。もし、どちらが当選しそうかはっきりとは言えない場合でも、どちらか一方、あえていえばより当選しそうと思われる方を選んでください。)
  Choice: Person 1 (人物１); Person 2 (人物2)

- (Preference, local) a Single Member District member of the House of Representatives (小選挙区選出の衆議院議員) replaced with a member of municipal council (市区町村議会議員).

- (Expectation, local) a Single Member District election of the House of Representatives (衆議院議員選挙の小選挙区) replaced with a municipal council election (市区町村議会議員選挙).

Table A2: Experiment 2 attributes and levels

Attribute	Levels
Party affiliation (所属政党)	Ruling party in the Diet (国会における与党);<br>Opposition party in the Diet (国会における野党);<br>No affiliation/minor party (無所属・諸派)
Gender (性別)	Male (男性); Female (女性)
Age (年齢)	35; 45; 55; 65
Educational attainment (最終学歴)	High school (高校卒); University (大学卒);<br>Graduate school (大学院卒)
Political experience (政治経験)	No experience (経験なし);<br>Incumbent (1 term) (現職（１期）);<br>Incumbent (2 terms) (現職（２期）);<br>Incumbent (5 terms) (現職（５期）);<br>Former (1 term) (元職（１期）)
Family structure (家族構成)	Single & have children (独身で子がいる);<br>Single & no children (独身で子はいない);<br>Married & have children (結婚して子がいる);<br>Married & no children (結婚して子はいない)
Living condition (居住状況)	Live with parents (親と共に居住);<br>Live close to parents (親の近くに居住);<br>Live far from parents (親から遠くに居住)
Policy focus (重点政策分野)	Defense/foreign policy (防衛・外交);<br>Safety/social order (治安・社会秩序);<br>Economy (経済); Welfare (福祉);<br>Environment (環境); Education (教育);<br>Women's status and social advancement (女性の地位・社会進出);<br>Local governance (地方自治)
Native Place (出身地)	Local municipality (地元の市区町村);<br>Local prefecture (地元の都道府県);<br>Non-local prefecture (地元ではない都道府県)

B Full Results of Conjoint Experiments

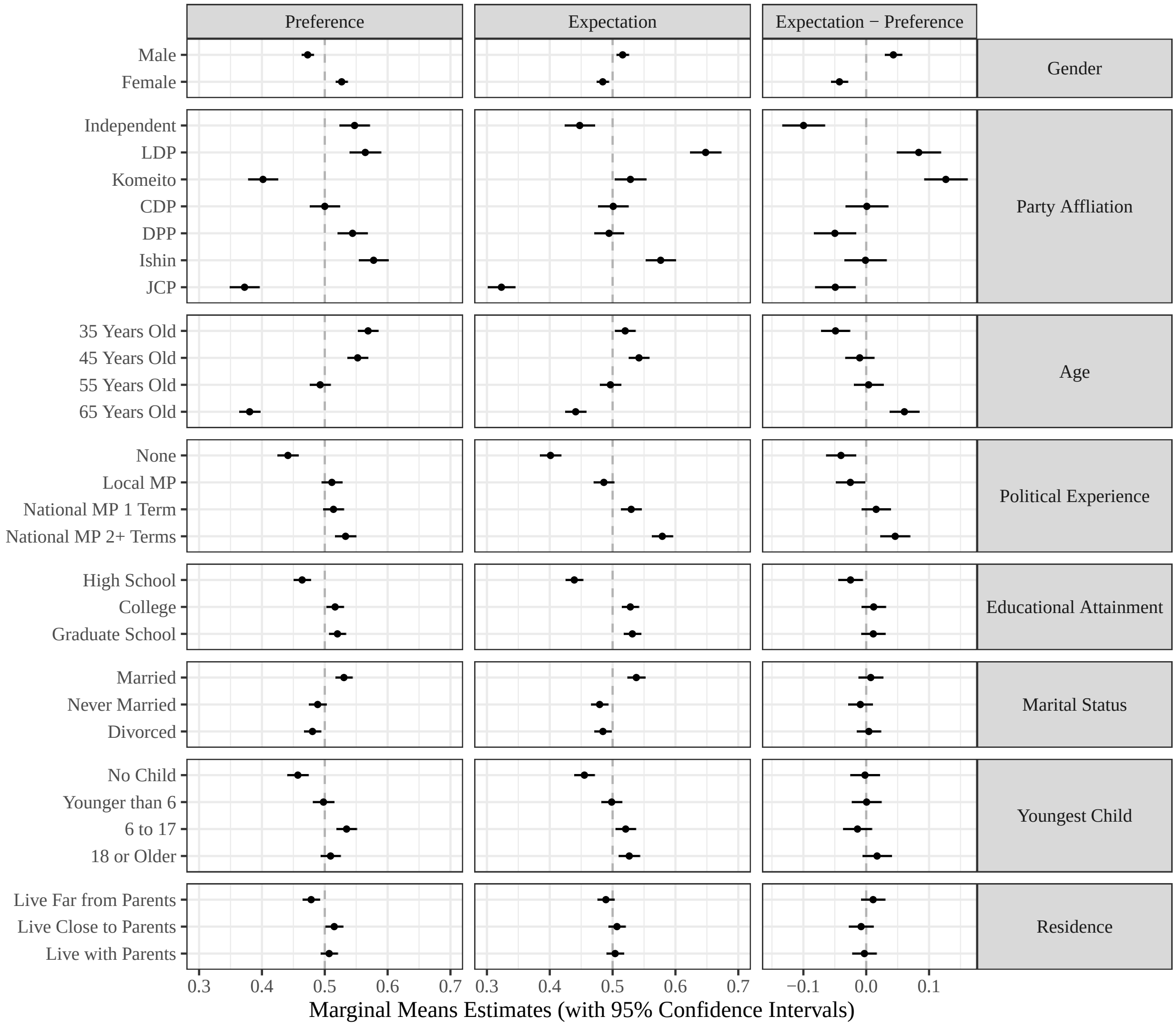

Figure A1: Full results from Experiment 1

		Preference	Expectation	Expectation − Preference
Gender	Male	0.000	0.001	0.000
	Female	0.000	0.001	0.000
Party Affliation	Independent	0.000	0.000	0.000
	LDP	0.000	0.000	0.000
	Komeito	0.000	0.026	0.000
	CDP	1.000	0.937	0.955
	DPP	0.000	0.633	0.003
	Ishin	0.000	0.000	0.943
	JCP	0.000	0.000	0.003
Age	35 Years Old	0.000	0.016	0.000
	45 Years Old	0.000	0.000	0.378
	55 Years Old	0.380	0.683	0.747
	65 Years Old	0.000	0.000	0.000
Political Experience	None	0.000	0.000	0.001
	Local MP	0.177	0.092	0.032
	National MP 1 Term	0.096	0.000	0.179
	National MP 2+ Terms	0.000	0.000	0.000
Educational Attainment	High School	0.000	0.000	0.011
	College	0.019	0.000	0.226
	Graduate School	0.003	0.000	0.247
Marital Status	Married	0.000	0.000	0.467
	Never Married	0.109	0.003	0.343
	Divorced	0.004	0.026	0.669
Youngest Child	No Child	0.000	0.000	0.867
	Younger than 6	0.807	0.856	0.961
	6 to 17	0.000	0.011	0.231
	18 or Older	0.253	0.002	0.141
Residence	Live Far from Parents	0.002	0.116	0.259
	Live Close to Parents	0.035	0.315	0.414
	Live with Parents	0.312	0.565	0.765

p−values from significance test

Note: H0=0.5 for preference and expectation tasks. H0=0 for preference−expecation gap.

Figure A2: p-values from significance tests in full results from Experiment 1

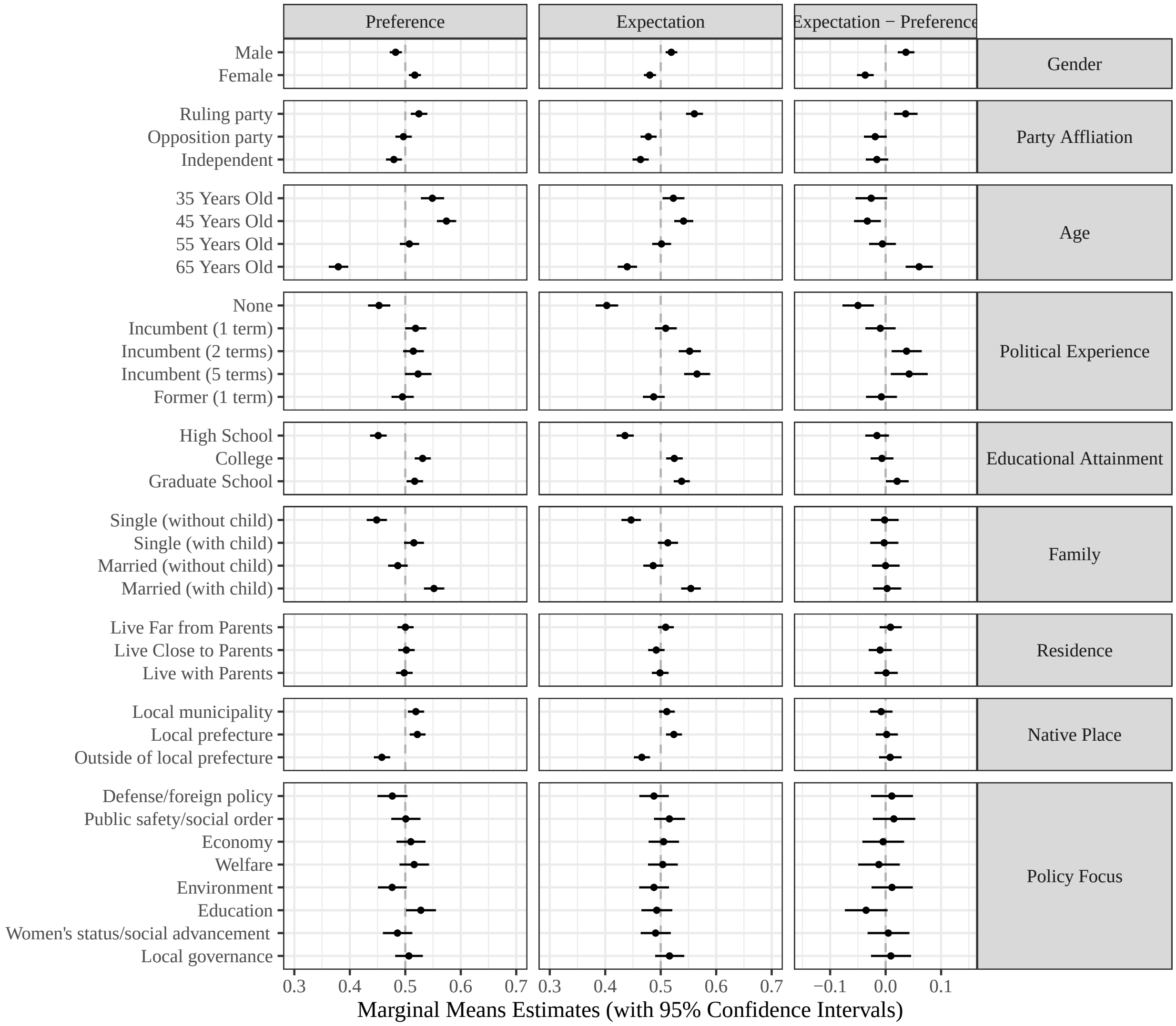

Figure A3: Full results from Experiment 2 (House of Representatives)

	Preference	Expectation	Expectation − Preference	
Male	0.001	0.000	0.000	Gender
Female	0.001	0.000	0.000	
Ruling party	0.001	0.000	0.001	Party Affliation
Opposition party	0.645	0.002	0.065	
Independent	0.003	0.000	0.118	
35 Years Old	0.000	0.020	0.069	Age
45 Years Old	0.000	0.000	0.006	
55 Years Old	0.401	0.871	0.627	
65 Years Old	0.000	0.000	0.000	
None	0.000	0.000	0.000	Political Experience
Incumbent (1 term)	0.052	0.358	0.488	
Incumbent (2 terms)	0.122	0.000	0.006	
Incumbent (5 terms)	0.054	0.000	0.012	
Former (1 term)	0.615	0.193	0.584	
High School	0.000	0.000	0.143	Educational Attainment
College	0.000	0.001	0.512	
Graduate School	0.021	0.000	0.044	
Single (without child)	0.000	0.000	0.884	Family
Single (with child)	0.083	0.157	0.836	
Married (without child)	0.122	0.127	0.995	
Married (with child)	0.000	0.000	0.838	
Live Far from Parents	0.983	0.202	0.379	Residence
Live Close to Parents	0.804	0.265	0.336	
Live with Parents	0.785	0.866	0.945	
Local municipality	0.009	0.121	0.424	Native Place
Local prefecture	0.002	0.001	0.853	
Outside of local prefecture	0.000	0.000	0.420	
Defense/foreign policy	0.086	0.358	0.559	Policy Focus
Public safety/social order	0.951	0.266	0.442	
Economy	0.449	0.699	0.808	
Welfare	0.232	0.783	0.518	
Environment	0.069	0.363	0.539	
Education	0.038	0.603	0.069	
Women's status/social advancement	0.288	0.496	0.797	
Local governance	0.605	0.228	0.604	

p−values from significance test

Note: H0=0.5 for preference and expectation tasks. H0=0 for preference−expecation gap.

Figure A4: p-values from significance tests in full results from Experiment 2 (House of Representatives)

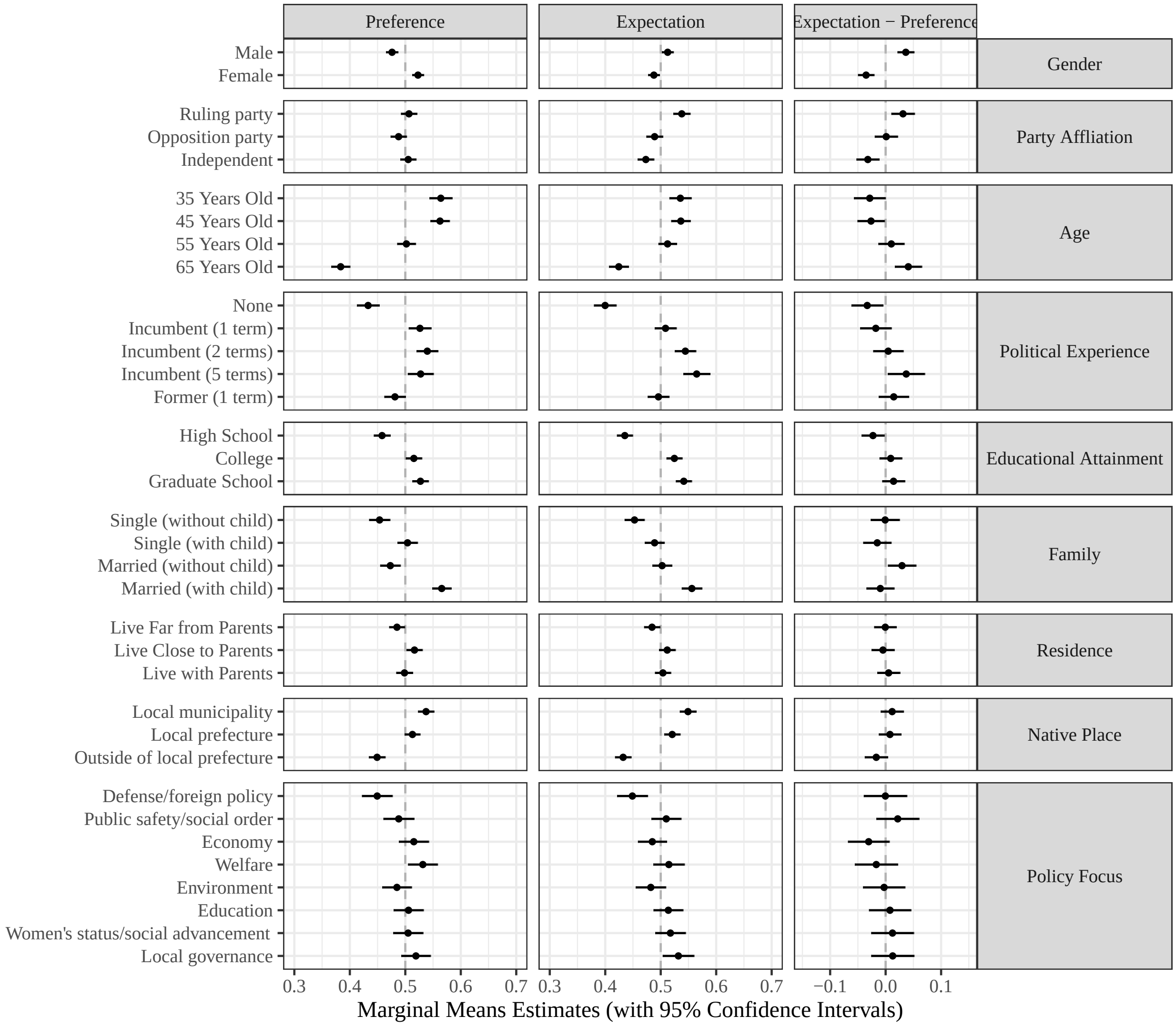

Figure A5: Full results from Experiment 2 (Municipal Council)

	Preference	Expectation	Expectation − Preference	
Male	0.000	0.018	0.000	Gender
Female	0.000	0.018	0.000	
Ruling party	0.364	0.000	0.003	Party Affliation
Opposition party	0.099	0.145	0.919	
Independent	0.469	0.000	0.002	
35 Years Old	0.000	0.000	0.049	Age
45 Years Old	0.000	0.000	0.034	
55 Years Old	0.810	0.141	0.387	
65 Years Old	0.000	0.000	0.001	
None	0.000	0.000	0.022	Political Experience
Incumbent (1 term)	0.010	0.384	0.217	
Incumbent (2 terms)	0.000	0.000	0.729	
Incumbent (5 terms)	0.019	0.000	0.029	
Former (1 term)	0.054	0.678	0.289	
High School	0.000	0.000	0.029	Educational Attainment
College	0.036	0.001	0.372	
Graduate School	0.000	0.000	0.168	
Single (without child)	0.000	0.000	0.948	Family
Single (with child)	0.660	0.217	0.239	
Married (without child)	0.004	0.776	0.022	
Married (with child)	0.000	0.000	0.455	
Live Far from Parents	0.035	0.031	0.950	Residence
Live Close to Parents	0.023	0.118	0.645	
Live with Parents	0.845	0.586	0.604	
Local municipality	0.000	0.000	0.261	Native Place
Local prefecture	0.068	0.005	0.442	
Outside of local prefecture	0.000	0.000	0.109	
Defense/foreign policy	0.000	0.000	0.982	Policy Focus
Public safety/social order	0.401	0.464	0.266	
Economy	0.262	0.249	0.108	
Welfare	0.020	0.305	0.391	
Environment	0.262	0.195	0.885	
Education	0.669	0.312	0.683	
Women's status/social advancement	0.709	0.209	0.526	
Local governance	0.154	0.027	0.518	

p−values from significance test

Note: H0=0.5 for preference and expectation tasks. H0=0 for preference−expecation gap.

Figure A6: p-values from significance tests in full results from Experiment 2 (Municipal Council)

C Main Results using Average Marginal Component Effect

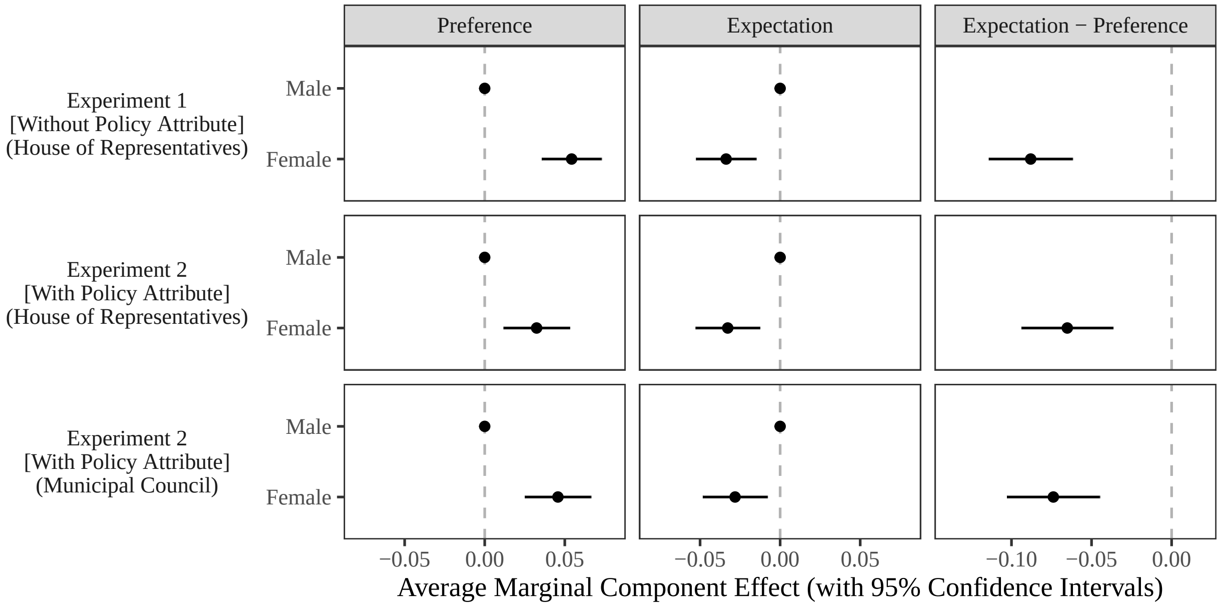

Figure A7: Individuals prefer female political candidates more than male ones, but they expect females to have lower chances to win elections than males (results using Average Marginal Component Effects)

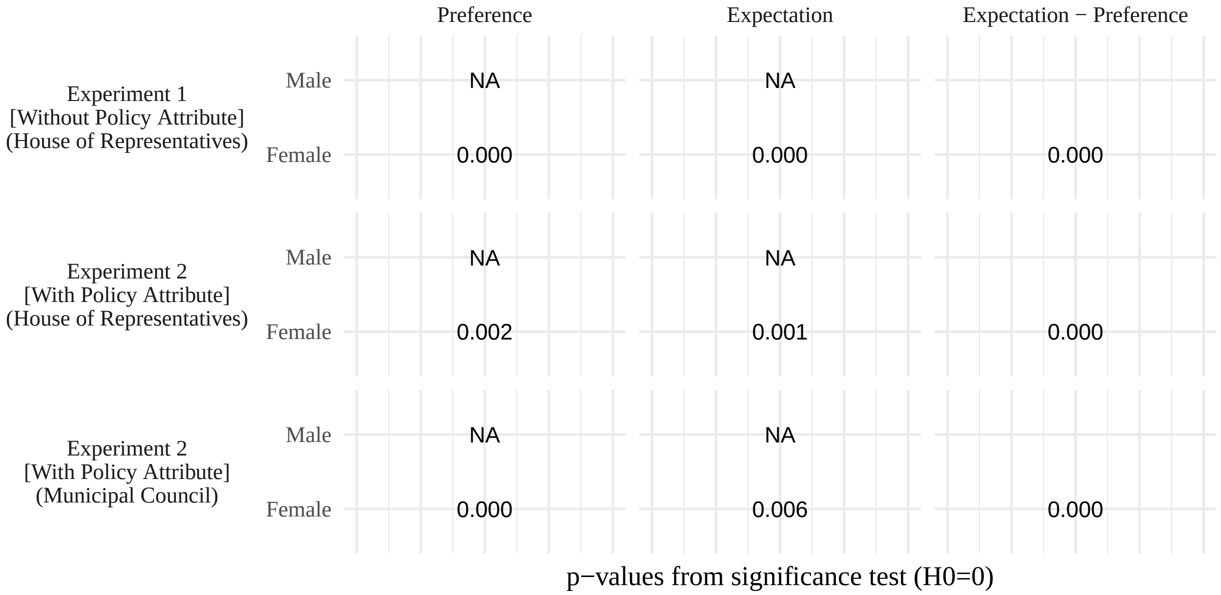

Figure A8: p-values for the main results using Average Marginal Component Effects

D Moderation by Gender Role Attitudes

D.1 The Set of Questions Used to Measure Gender Role Attitudes

Q: There are the following opinions on the state of men and women in society. Please tell us how you feel about each of them. (社会における男性と女性のあり方について、次のような意見があります。それぞれについてのあなたのお気持ちをお答えください。)

A: Disagree (反対) (1); Rather disagree (どちらかといえば反対) (2); Rather agree (どちらかといえば賛成) (3); Agree (賛成) (4)

1. If you are not satisfied with your partner after marriage, you can always divorce (結婚しても、相手に満足できないときは、いつでも離婚すればよい)
2. If the husband has enough income, the wife should not have a job (夫に充分な収入がある場合には、妻は仕事をもたない方がよい)
3. After all, a woman's happiness lies in marriage (なんといっても女性の幸福は結婚にある)
4. Men should also be responsible for personal care and cooking (男性も身の回りのことや炊事をすべきだ)
5. Husbands should work outside the home and wives should protect the home (夫は外で働き、妻は家庭を守るべきだ)
6. After all, a man's happiness lies in marriage (なんといっても男性の幸福は結婚にある)
7. Mothers who work have a negative impact on their children before they enter elementary school (母親が仕事をもつと、小学校へあがる前の子どもによくない影響を与える)
8. It is not necessary to have children after marriage (結婚しても、必ずしも子どもをもつ必要はない)
9. It is more important for a wife to help her husband with his work than to have a job of her own (妻にとっては、自分の仕事をもつよりも、夫の仕事の手助けをする方が大切である)

We use the following procedure to generate a binary variable of gender role attitudes:

1. Impute missing values by the intermediate score (= 2.5).
2. Pool respondents in Experiments 1 and 2 and perform a factor analysis with a single factor principal factor solution.
3. Calculate the factor score using the Bartlett method.
4. Identify the median of the factor score (pooling respondents in Experiments 1 and 2) and split each group of respondents by this median value.

D.2 Results using Average Marginal Component Effect

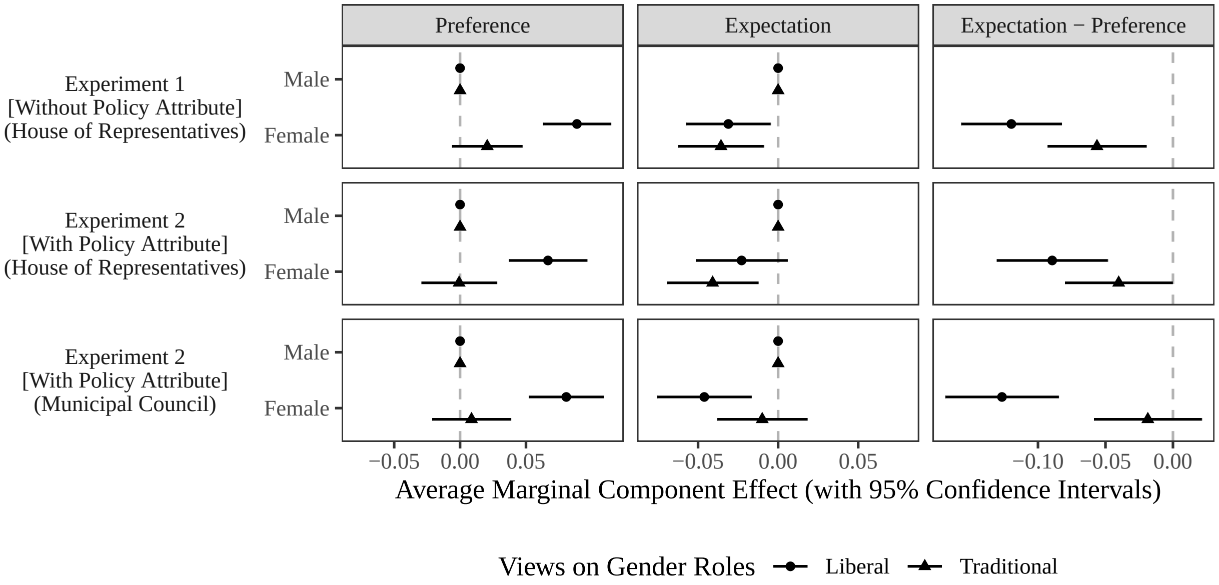

Figure A9: Preference-expectation gap is larger for those who have a liberal gender role view, because they prefer women more strongly but expect women to be no more advantaged compared to those who have a traditional gender role view

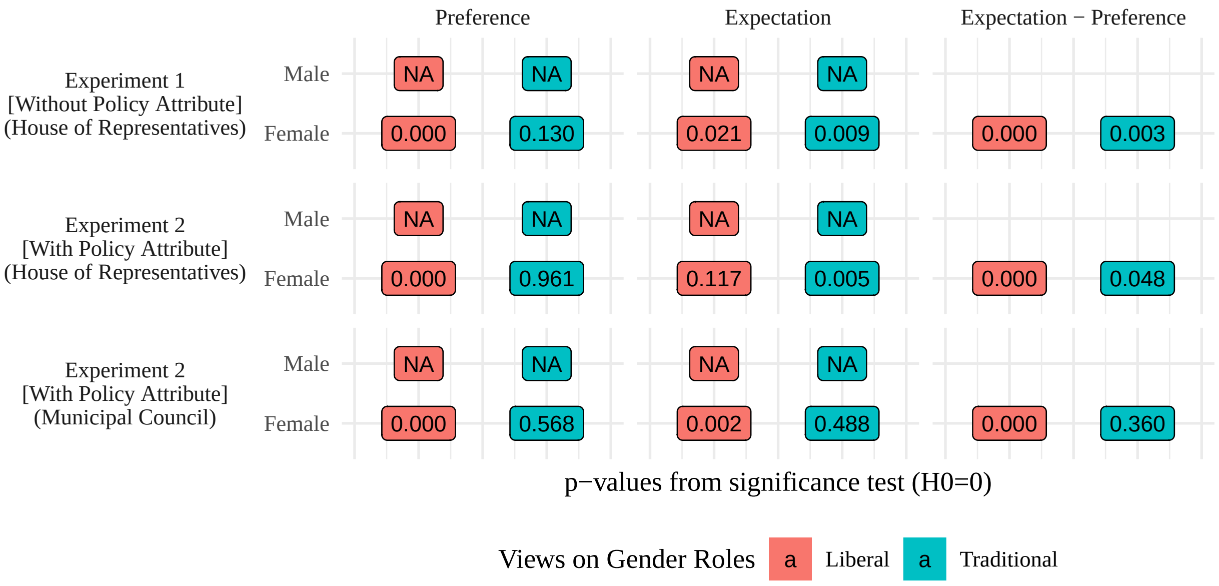

Figure A10: p-values from the results on the moderating effect of gender role attitudes using Average Marginal Component Effect

D.3 Full Results of Conjoint Experiments

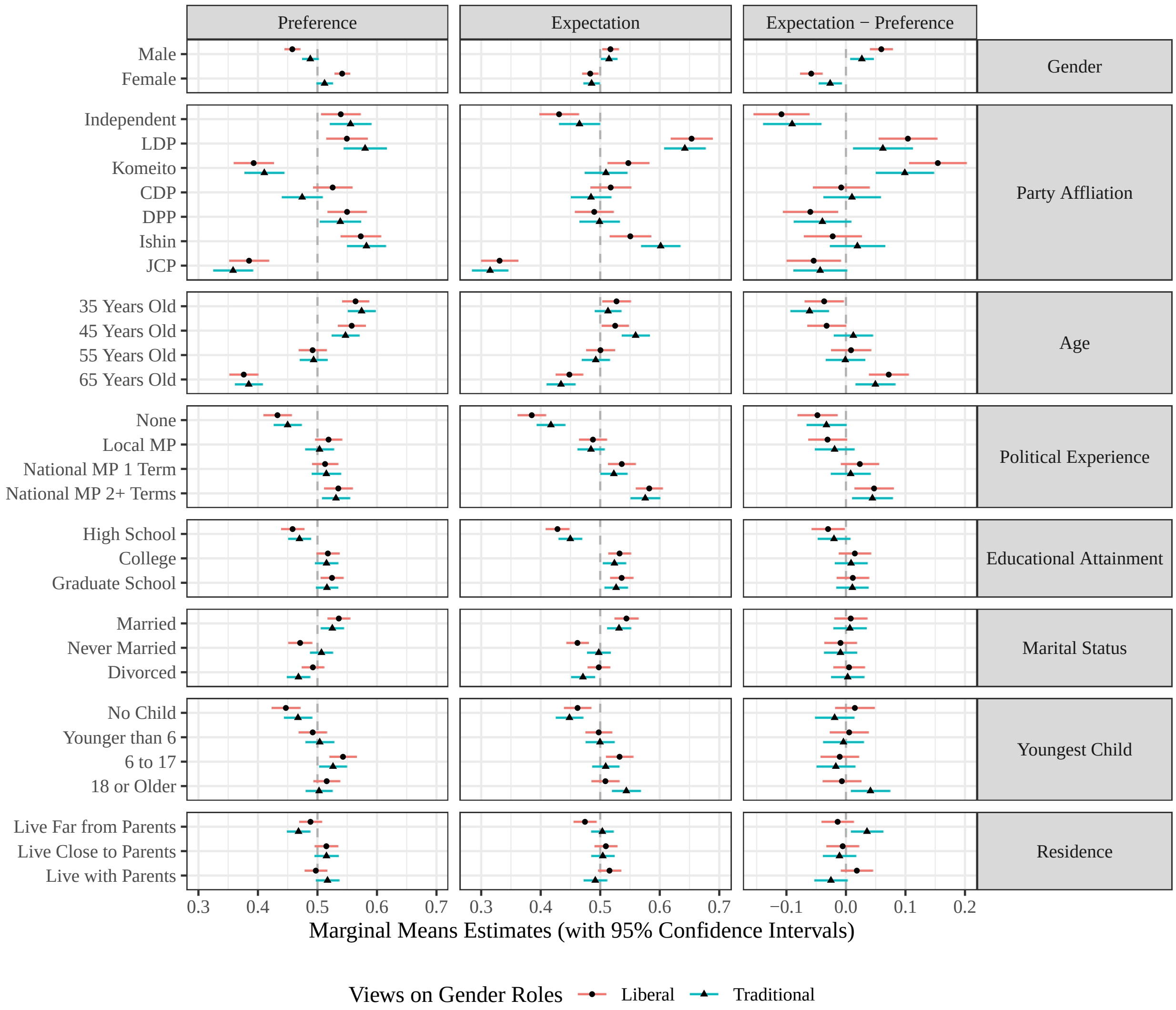

Figure A11: Full results from Experiment 1, moderated by gender role attitudes

Group	Attribute	Preference (Liberal)	Preference (Traditional)	Expectation (Liberal)	Expectation (Traditional)	Expectation − Preference (Liberal)	Expectation − Preference (Traditional)
Gender	Male	0.000	0.086	0.014	0.036	0.000	0.007
	Female	0.000	0.085	0.014	0.035	0.000	0.007
Party Affliation	Independent	0.020	0.002	0.000	0.045	0.000	0.000
	LDP	0.005	0.000	0.000	0.000	0.000	0.015
	Komeito	0.000	0.000	0.008	0.599	0.000	0.000
	CDP	0.128	0.139	0.312	0.367	0.742	0.678
	DPP	0.003	0.029	0.541	0.941	0.011	0.106
	Ishin	0.000	0.000	0.004	0.000	0.367	0.413
	JCP	0.000	0.000	0.000	0.000	0.019	0.059
Age	35 Years Old	0.000	0.000	0.024	0.249	0.028	0.000
	45 Years Old	0.000	0.000	0.029	0.000	0.049	0.459
	55 Years Old	0.493	0.580	0.975	0.527	0.616	0.949
	65 Years Old	0.000	0.000	0.000	0.000	0.000	0.004
Political Experience	None	0.000	0.000	0.000	0.000	0.005	0.053
	Local MP	0.104	0.775	0.300	0.178	0.061	0.257
	National MP 1 Term	0.250	0.230	0.002	0.047	0.150	0.640
	National MP 2+ Terms	0.004	0.009	0.000	0.000	0.005	0.010
Educational Attainment	High School	0.000	0.002	0.000	0.000	0.032	0.148
	College	0.075	0.125	0.001	0.015	0.276	0.537
	Graduate School	0.012	0.091	0.000	0.007	0.403	0.431
Marital Status	Married	0.000	0.011	0.000	0.002	0.563	0.640
	Never Married	0.004	0.492	0.000	0.801	0.509	0.510
	Divorced	0.417	0.001	0.796	0.004	0.697	0.836
Youngest Child	No Child	0.000	0.006	0.001	0.000	0.374	0.256
	Younger than 6	0.507	0.754	0.823	0.974	0.742	0.808
	6 to 17	0.000	0.027	0.005	0.427	0.524	0.304
	18 or Older	0.172	0.815	0.464	0.000	0.677	0.014
Residence	Live Far from Parents	0.226	0.001	0.008	0.711	0.309	0.010
	Live Close to Parents	0.134	0.137	0.329	0.661	0.695	0.444
	Live with Parents	0.772	0.090	0.113	0.403	0.180	0.074

p−values from significance test

Views on Gender Roles: Liberal, Traditional

Note: H0=0.5 for preference and expectation tasks. H0=0 for preference−expecation gap.

Figure A12: p-values from significance tests in full results from Experiment 1, moderated by gender role attitudes

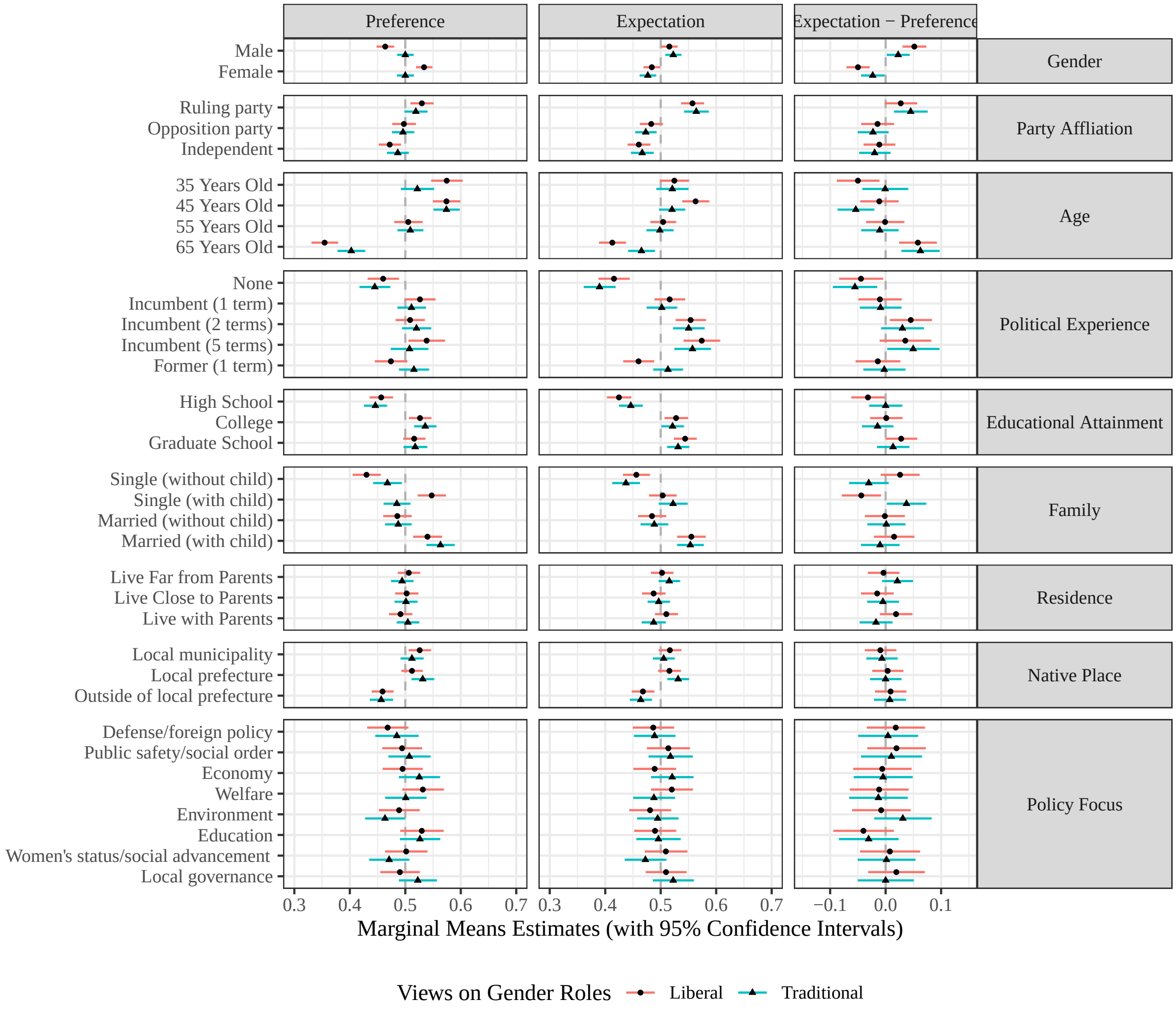

Figure A13: Full results from Experiment 2 (House of Representatives), moderated by gender role attitudes

		Preference		Expectation		Expectation − Preference	
		Liberal	Traditional	Liberal	Traditional	Liberal	Traditional
Gender	Male	0.000	1.000	0.033	0.002	0.000	0.028
	Female	0.000	1.000	0.033	0.002	0.000	0.028
Party Affliation	Ruling party	0.004	0.068	0.000	0.000	0.062	0.003
	Opposition party	0.815	0.676	0.102	0.005	0.329	0.103
	Independent	0.005	0.169	0.000	0.001	0.428	0.163
Age	35 Years Old	0.000	0.149	0.062	0.152	0.010	0.969
	45 Years Old	0.000	0.000	0.000	0.086	0.514	0.001
	55 Years Old	0.677	0.440	0.711	0.898	0.954	0.532
	65 Years Old	0.000	0.000	0.000	0.004	0.001	0.000
Political Experience	None	0.005	0.000	0.000	0.000	0.027	0.006
	Incumbent (1 term)	0.059	0.390	0.246	0.888	0.602	0.628
	Incumbent (2 terms)	0.515	0.127	0.000	0.000	0.018	0.120
	Incumbent (5 terms)	0.021	0.657	0.000	0.001	0.131	0.037
	Former (1 term)	0.078	0.254	0.004	0.334	0.490	0.898
Educational Attainment	High School	0.000	0.000	0.000	0.000	0.037	0.998
	College	0.009	0.000	0.009	0.037	0.934	0.308
	Graduate School	0.108	0.097	0.000	0.002	0.051	0.361
Family	Single (without child)	0.000	0.013	0.000	0.000	0.139	0.090
	Single (with child)	0.000	0.210	0.770	0.089	0.014	0.036
	Married (without child)	0.265	0.285	0.217	0.354	0.938	0.945
	Married (with child)	0.002	0.000	0.000	0.000	0.406	0.567
Residence	Live Far from Parents	0.535	0.566	0.813	0.112	0.789	0.130
	Live Close to Parents	0.813	0.907	0.227	0.715	0.305	0.735
	Live with Parents	0.412	0.651	0.329	0.241	0.204	0.243
Native Place	Local municipality	0.010	0.251	0.105	0.588	0.511	0.641
	Local prefecture	0.211	0.002	0.129	0.001	0.796	0.998
	Outside of local prefecture	0.000	0.000	0.002	0.000	0.531	0.607
Policy Focus	Defense/foreign policy	0.090	0.438	0.473	0.558	0.491	0.877
	Public safety/social order	0.749	0.705	0.485	0.381	0.465	0.710
	Economy	0.790	0.178	0.574	0.285	0.821	0.864
	Welfare	0.095	0.966	0.292	0.520	0.664	0.626
	Environment	0.551	0.044	0.314	0.769	0.763	0.237
	Education	0.134	0.150	0.592	0.833	0.147	0.261
	Women's status/social advancement	0.934	0.111	0.632	0.145	0.779	0.955
	Local governance	0.587	0.194	0.608	0.229	0.455	0.999

p−values from significance test

Views on Gender Roles: a Liberal, a Traditional

Note: H0=0.5 for preference and expectation tasks. H0=0 for preference−expecation gap.

Figure A14: p-values from significance tests in full results from Experiment 2 (House of Representatives), moderated by gender role attitudes

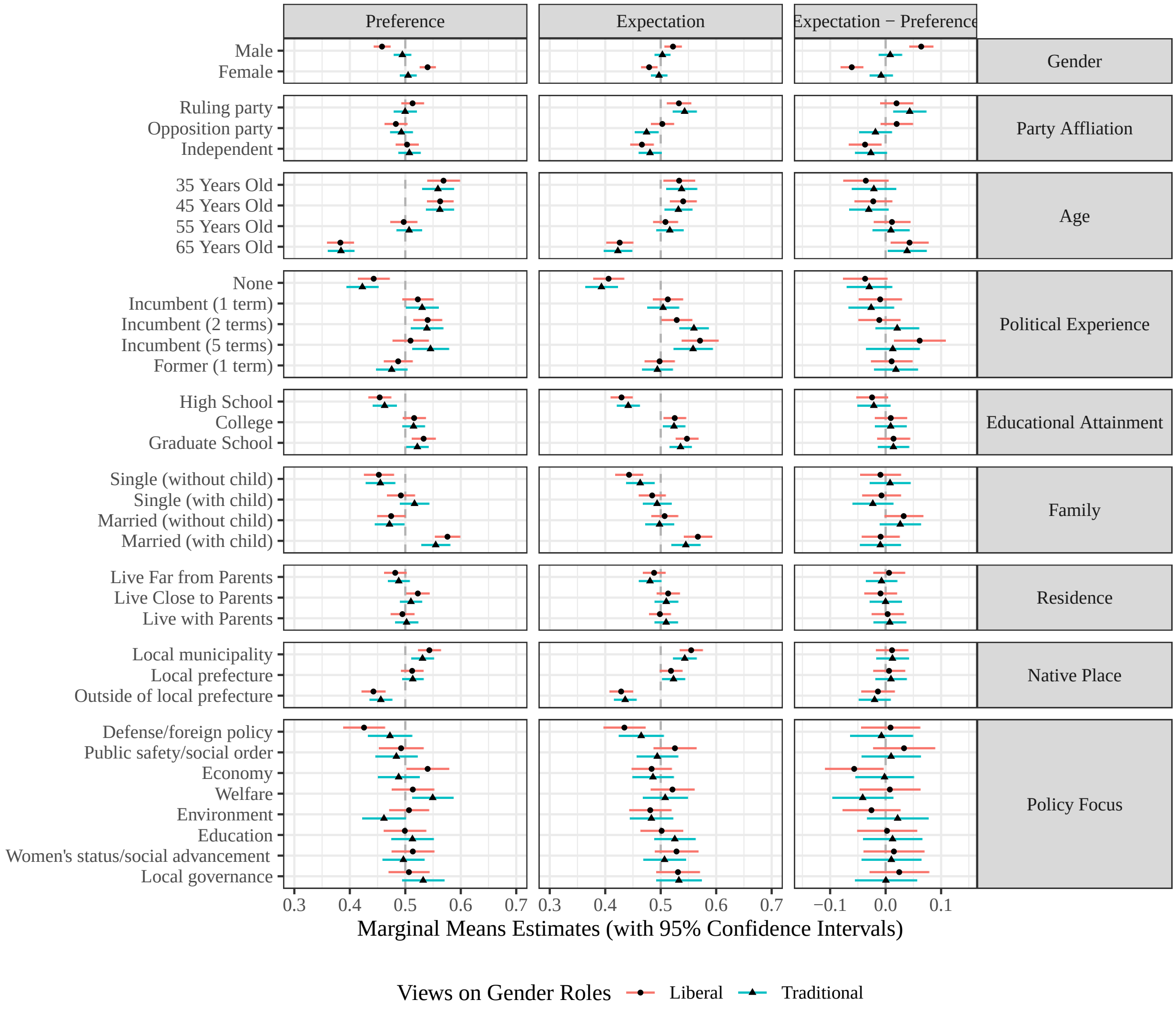

Figure A15: Full results from Experiment 2 (Municipal Council), moderated by gender role attitudes

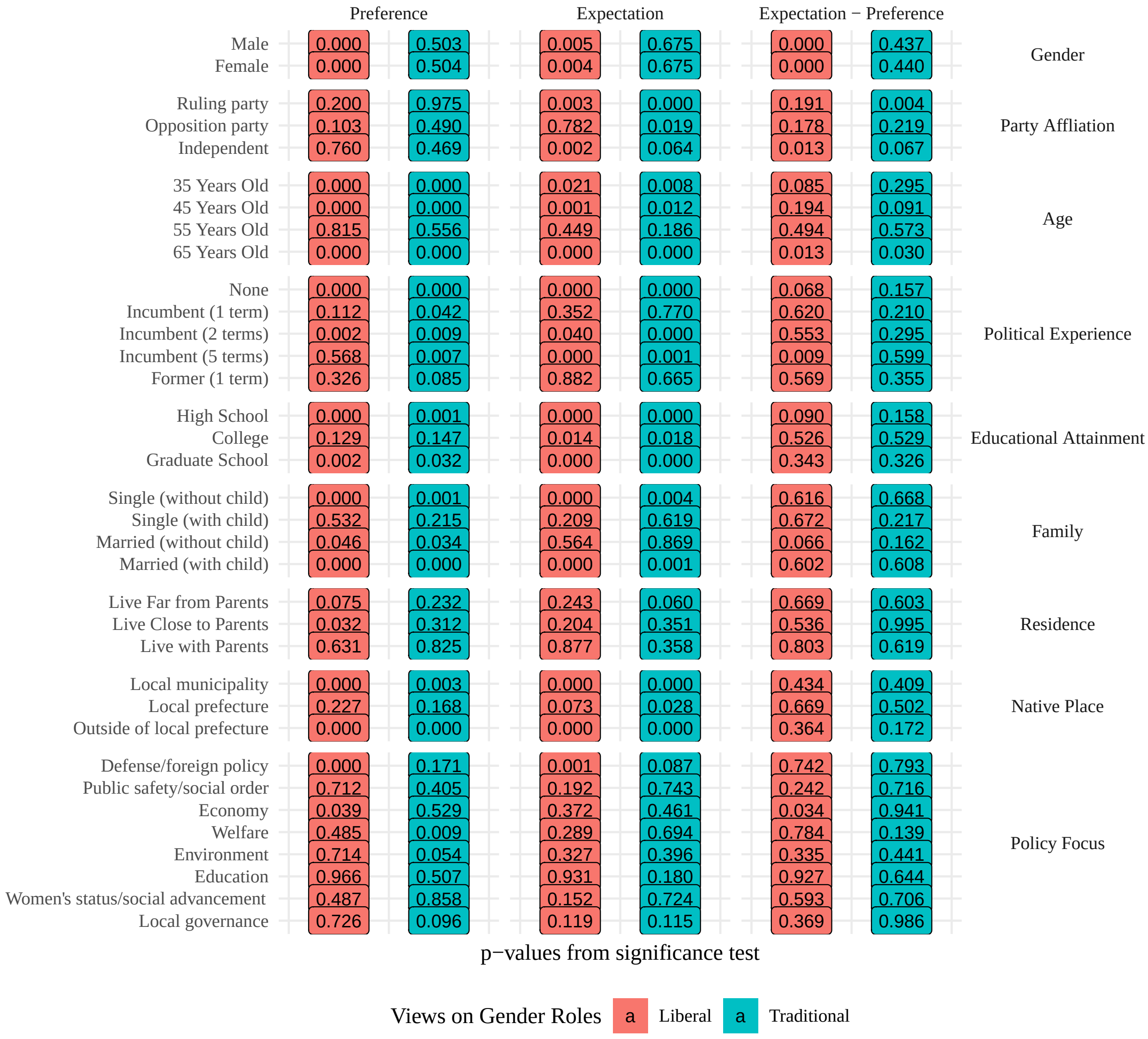

Note: H0=0.5 for preference and expectation tasks. H0=0 for preference−expecation gap.

Figure A16: p-values from significance tests in full results from Experiment 2 (Municipal Council), moderated by gender role attitudes

E Moderation by Respondent's Gender

Respondents with self-identified nonbinary gender (neither male nor female) are excluded from all the analyses below.

E.1 Figure that is parallel to Figure 2

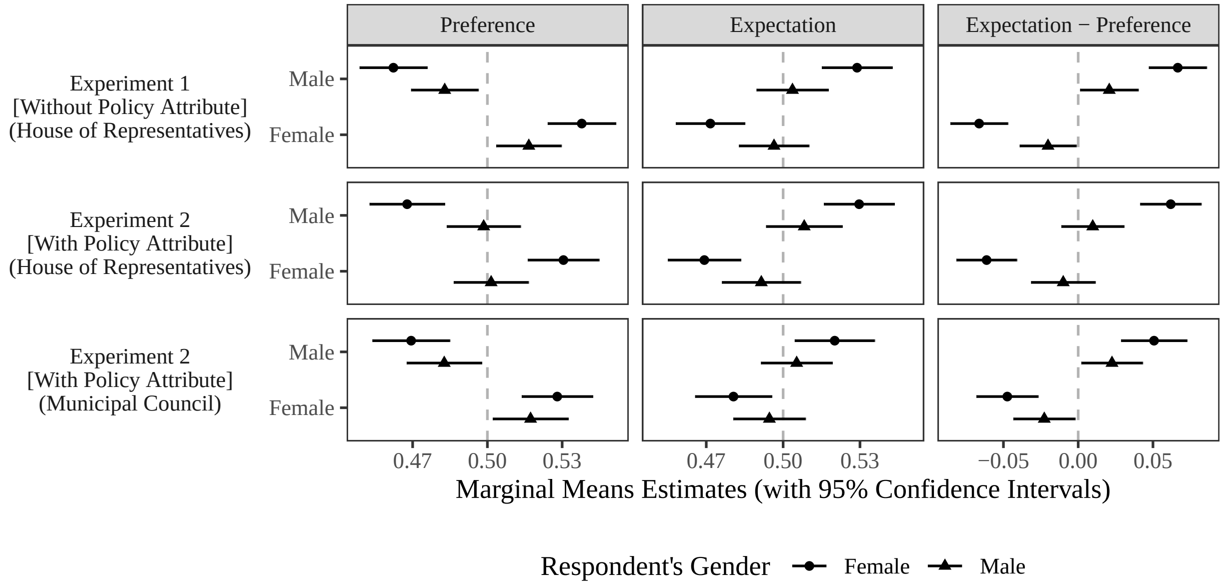

Figure A17: Preference-expectation gap in support for female candidates is larger for female voters, because they prefer women more strongly but expect women to be no more advantaged or even disadvantaged compared to male voters

E.2 Results using Average Marginal Component Effect

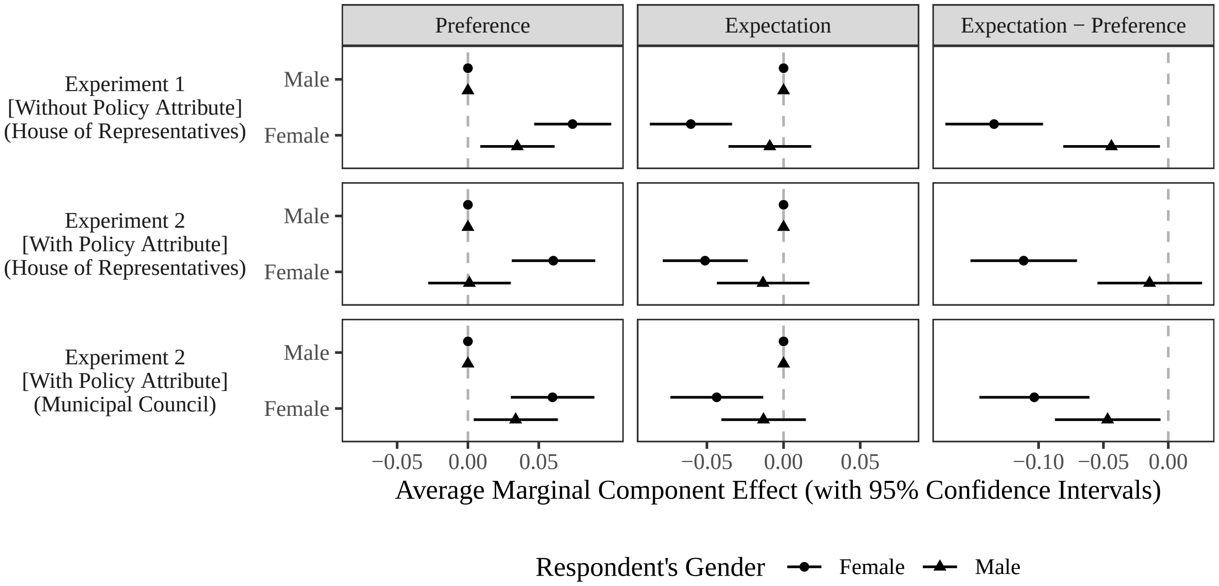

Figure A18: Results on the moderating effect of respondent's gender using Average Marginal Component Effect

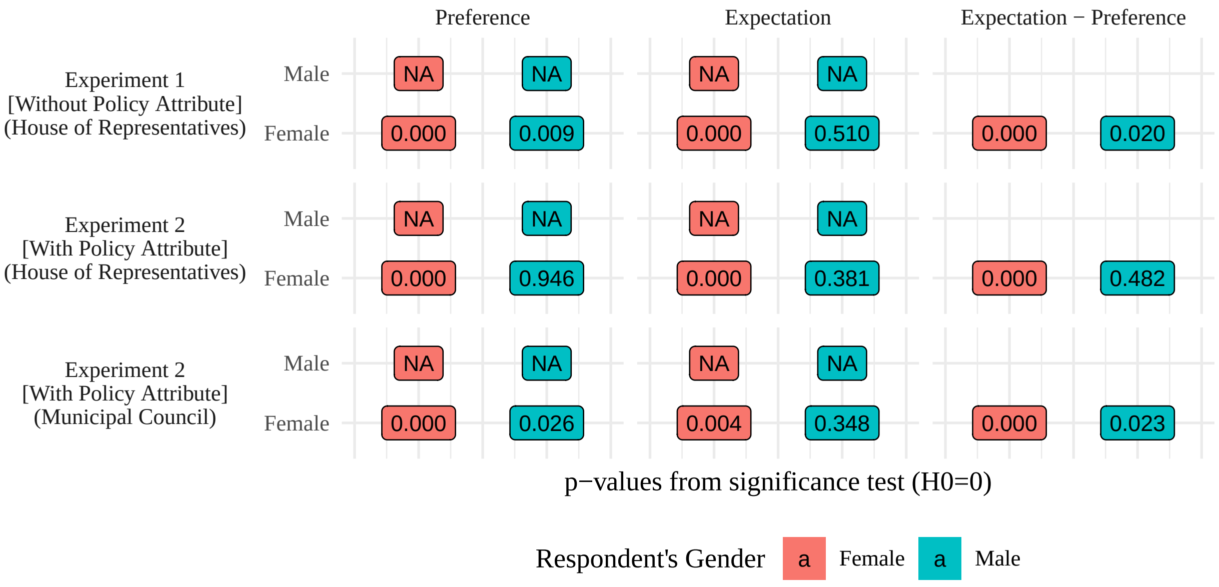

Figure A19: p-values from the results on the moderating effect of respondent's gender using Average Marginal Component Effect

E.3 Full Results of Conjoint Experiments

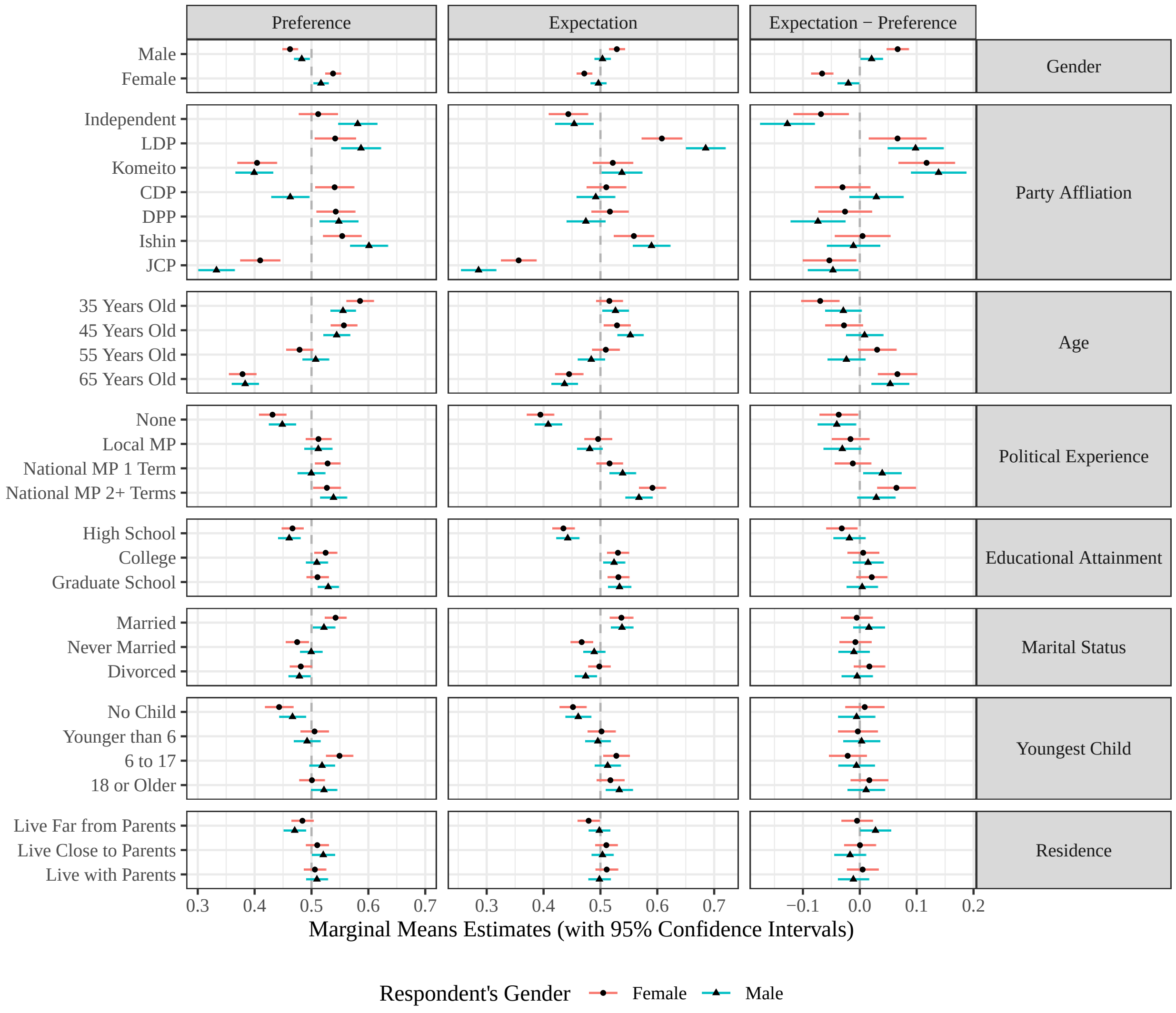

Figure A20: Full results from Experiment 1, moderated by respondent's gender

Attribute	Level	Preference: Female	Preference: Male	Expectation: Female	Expectation: Male	Expectation − Preference: Female	Expectation − Preference: Male
Gender	Male	0.000	0.013	0.000	0.610	0.000	0.036
	Female	0.000	0.013	0.000	0.610	0.000	0.036
Party Affliation	Independent	0.499	0.000	0.001	0.007	0.006	0.000
	LDP	0.024	0.000	0.000	0.000	0.010	0.000
	Komeito	0.000	0.000	0.227	0.037	0.000	0.000
	CDP	0.019	0.028	0.561	0.632	0.220	0.229
	DPP	0.014	0.005	0.313	0.138	0.278	0.003
	Ishin	0.002	0.000	0.001	0.000	0.846	0.636
	JCP	0.000	0.000	0.000	0.000	0.025	0.036
Age	35 Years Old	0.000	0.000	0.186	0.024	0.000	0.076
	45 Years Old	0.000	0.000	0.015	0.000	0.098	0.613
	55 Years Old	0.080	0.531	0.441	0.180	0.076	0.163
	65 Years Old	0.000	0.000	0.000	0.000	0.000	0.001
Political Experience	None	0.000	0.000	0.000	0.000	0.031	0.018
	Local MP	0.284	0.339	0.740	0.096	0.330	0.068
	National MP 1 Term	0.012	0.976	0.172	0.001	0.452	0.021
	National MP 2+ Terms	0.028	0.001	0.000	0.000	0.000	0.089
Educational Attainment	High School	0.001	0.000	0.000	0.000	0.022	0.200
	College	0.014	0.334	0.002	0.014	0.674	0.291
	Graduate School	0.284	0.002	0.001	0.001	0.127	0.763
Marital Status	Married	0.000	0.028	0.000	0.000	0.703	0.254
	Never Married	0.013	0.954	0.001	0.261	0.586	0.455
	Divorced	0.055	0.030	0.839	0.008	0.227	0.734
Youngest Child	No Child	0.000	0.005	0.000	0.001	0.618	0.732
	Younger than 6	0.668	0.512	0.877	0.686	0.845	0.846
	6 to 17	0.000	0.103	0.017	0.275	0.207	0.719
	18 or Older	0.946	0.059	0.155	0.006	0.317	0.506
Residence	Live Far from Parents	0.104	0.003	0.037	0.837	0.731	0.044
	Live Close to Parents	0.318	0.044	0.298	0.709	0.985	0.228
	Live with Parents	0.546	0.325	0.271	0.859	0.721	0.415

p−values from significance test

Respondent's Gender: Female, Male

Note: H0=0.5 for preference and expectation tasks. H0=0 for preference−expecation gap.

Figure A21: p-values from significance tests in full results from Experiment 1, moderated by respondent's gender

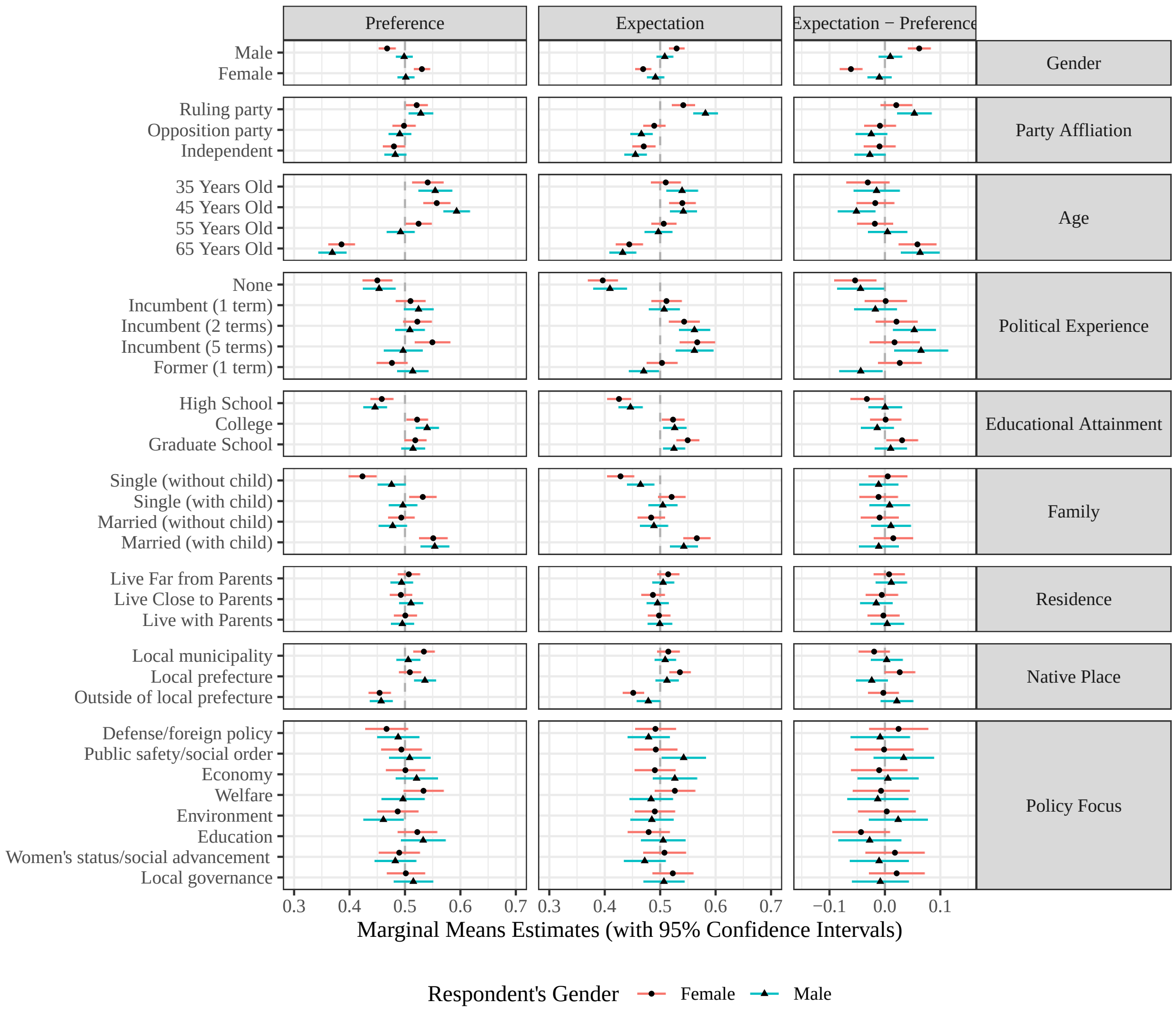

Figure A22: Full results from Experiment 2 (House of Representatives), moderated by respondent's gender

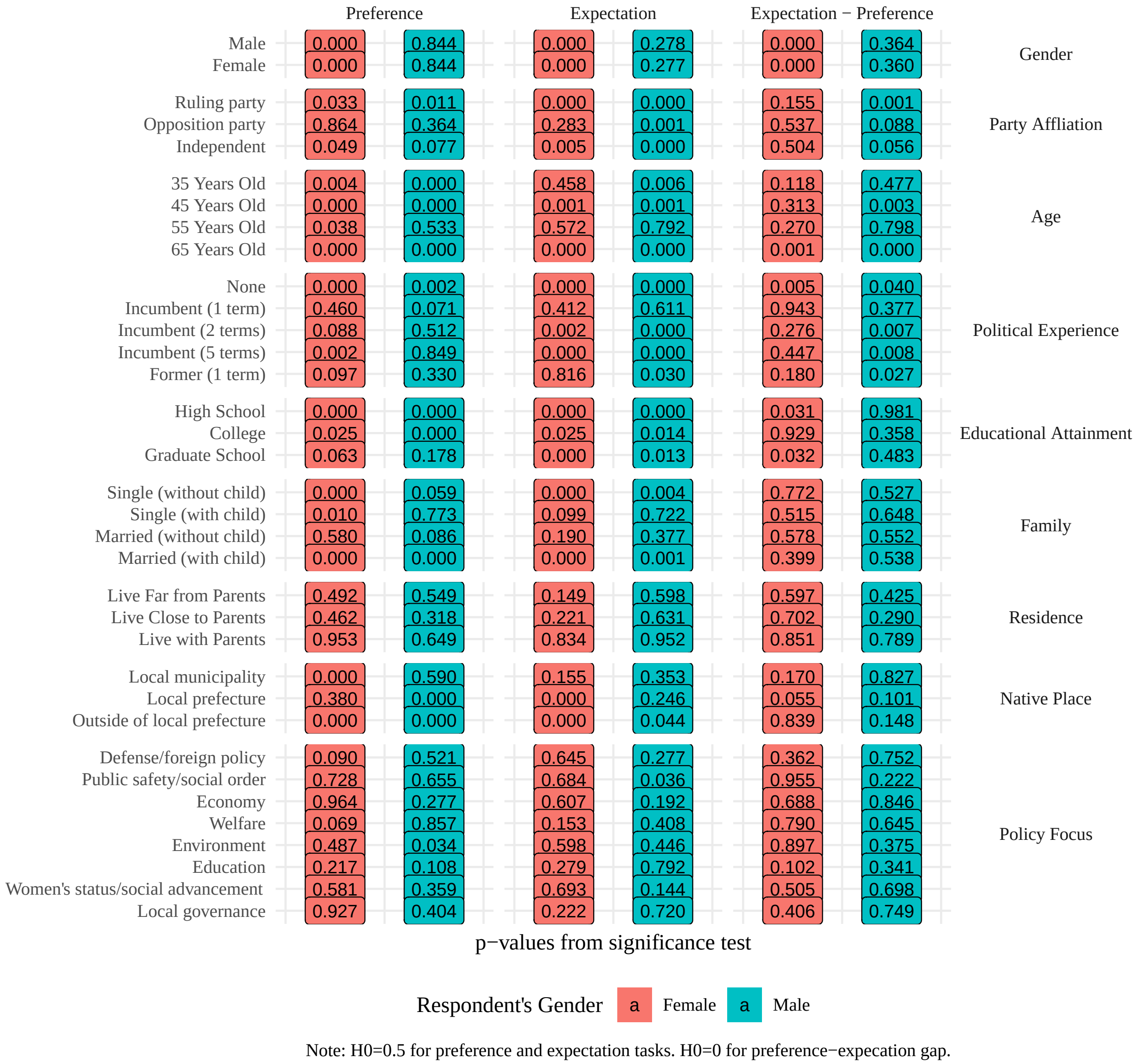

Figure A23: p-values from significance tests in full results from Experiment 2 (House of Representatives), moderated by respondent's gender

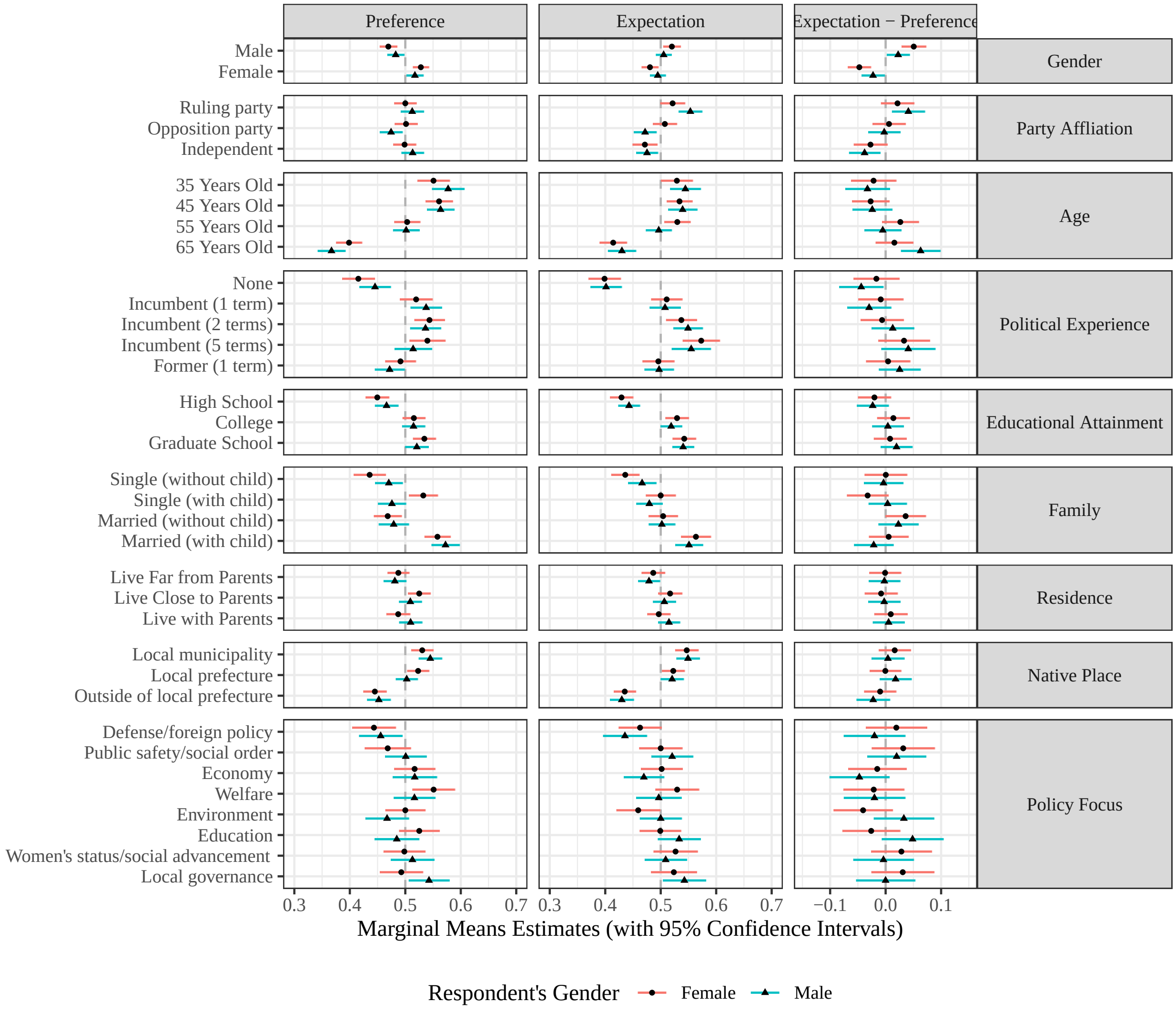

Figure A24: Full results from Experiment 2 (Municipal Council), moderated by respondent's gender

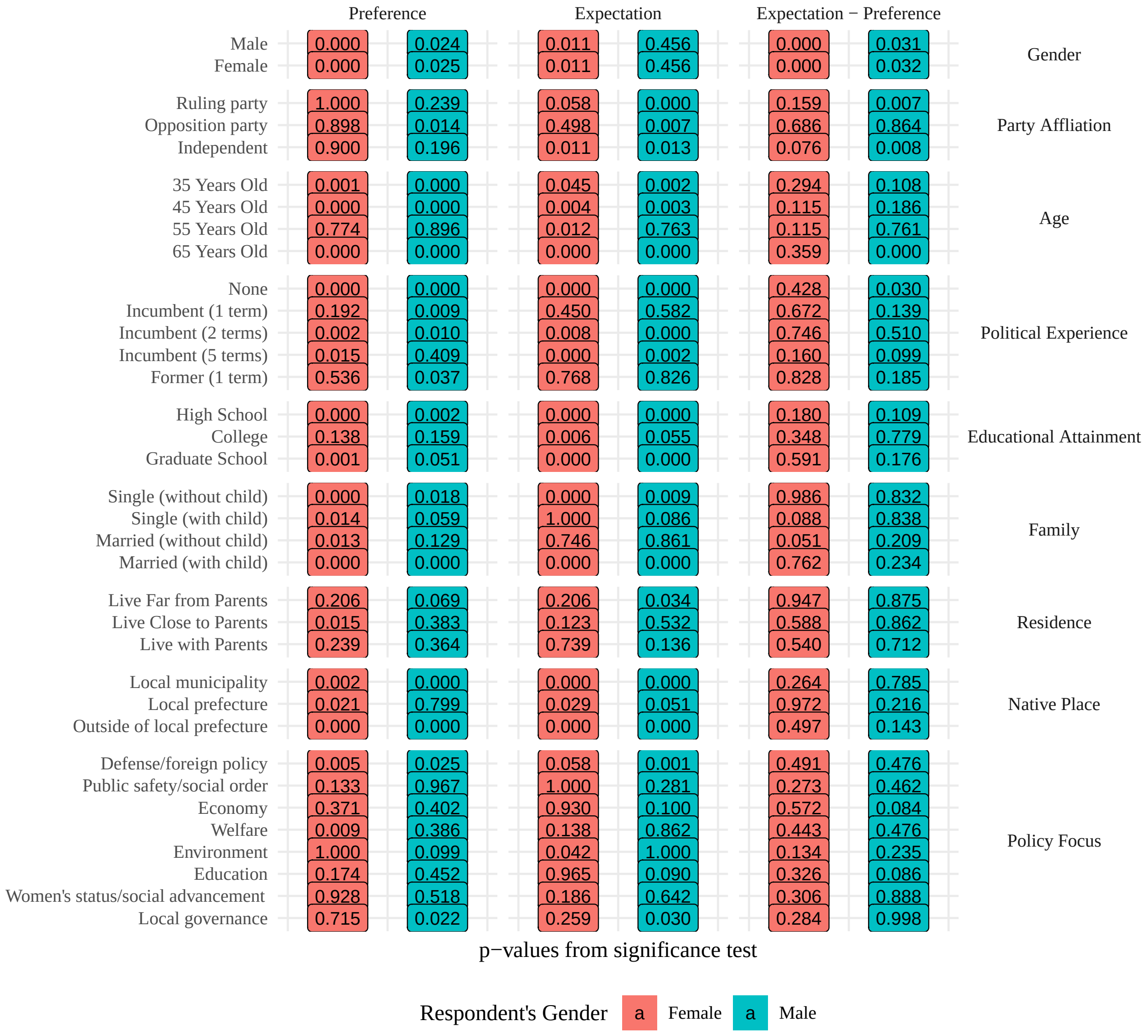

Note: H0=0.5 for preference and expectation tasks. H0=0 for preference−expecation gap.

Figure A25: p-values from significance tests in full results from Experiment 2 (Municipal Council), moderated by respondent's gender

F Moderation by Candidate's Party

F.1 Figure that is parallel to Figure 2

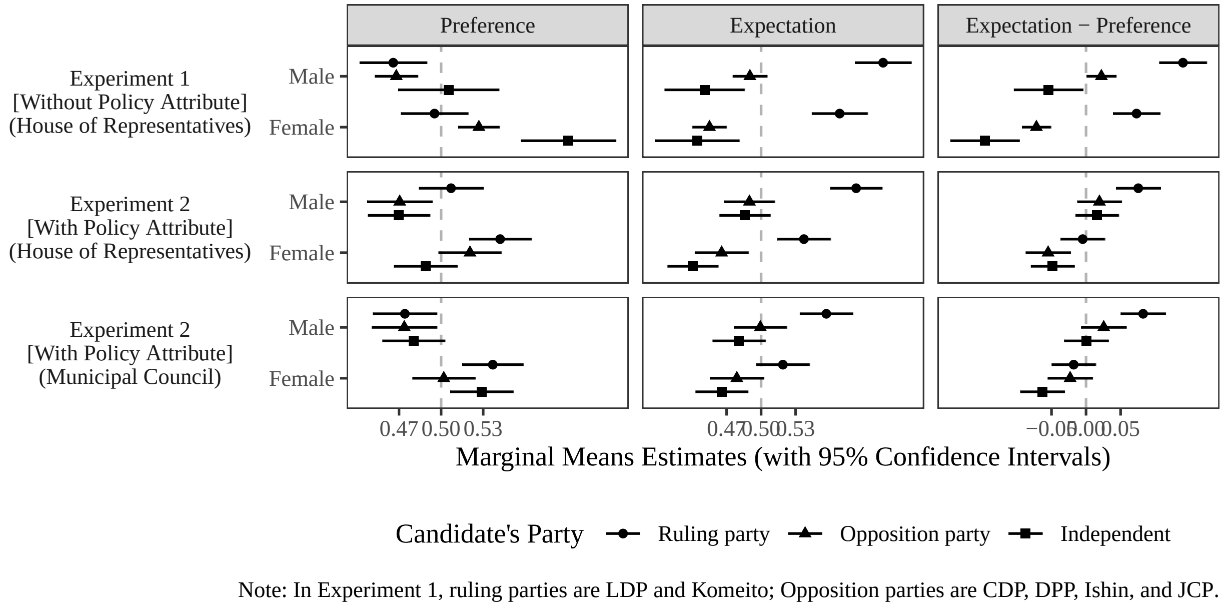

Figure A26: Preference-expectation gap in support for female candidates is no smaller for ruling party candidates after filtering away the direct effect of party status

F.2 Results using Average Marginal Component Effect

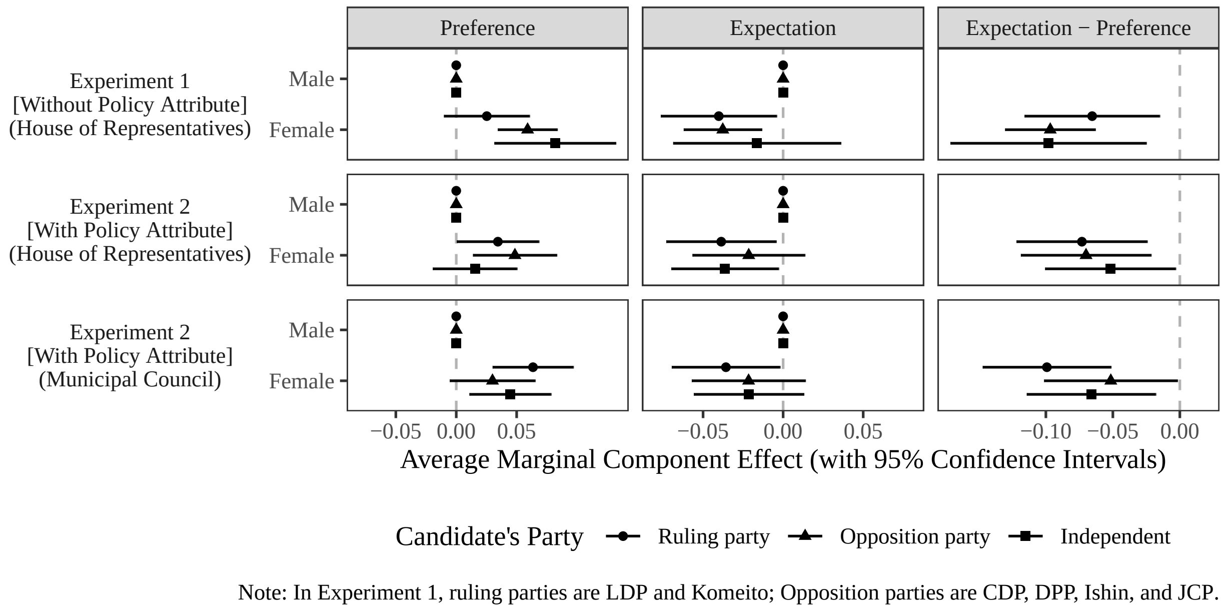

Figure A27: Results on the moderating effect of candidate's party using Average Marginal Component Effect

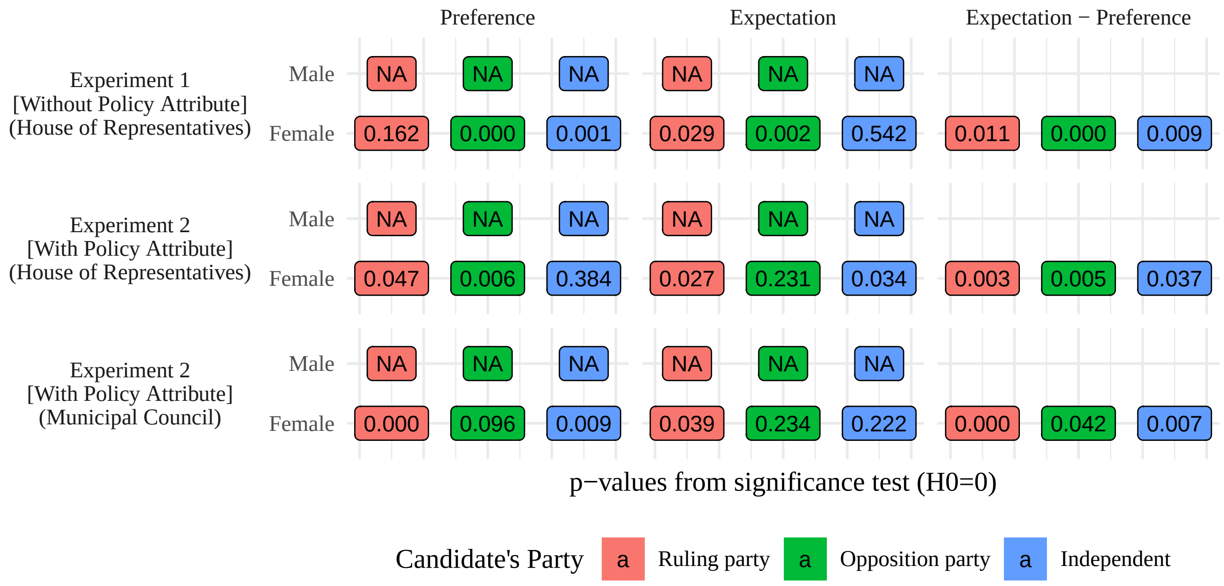

Figure A28: p-values from the results on the moderating effect of candidate's party using Average Marginal Component Effect

F.3 Full Results of Conjoint Experiments

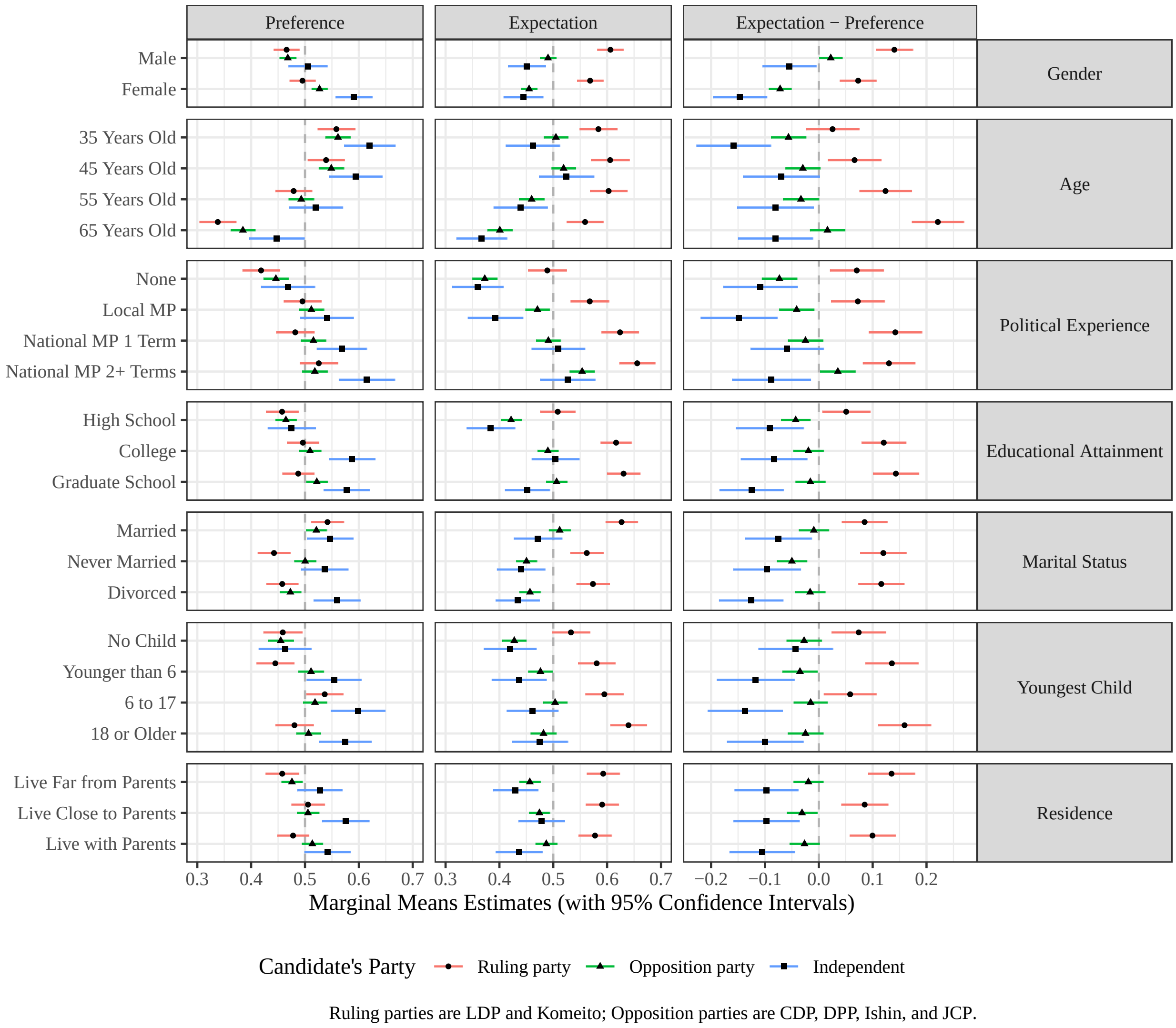

Figure A29: Full results from Experiment 1, moderated by candidate's party

		Preference			Expectation			Expectation − Preference		
		Ruling party	Opposition party	Independent	Ruling party	Opposition party	Independent	Ruling party	Opposition party	Independent
Gender	Male	0.005	0.000	0.772	0.000	0.201	0.006	0.000	0.042	0.033
Gender	Female	0.700	0.000	0.000	0.000	0.000	0.003	0.000	0.000	0.000
Age	35 Years Old	0.001	0.000	0.000	0.000	0.665	0.137	0.309	0.001	0.000
Age	45 Years Old	0.024	0.000	0.000	0.000	0.093	0.347	0.008	0.072	0.055
Age	55 Years Old	0.222	0.556	0.433	0.000	0.001	0.017	0.000	0.050	0.025
Age	65 Years Old	0.000	0.000	0.045	0.001	0.000	0.000	0.000	0.331	0.023
Political Experience	None	0.000	0.000	0.214	0.543	0.000	0.000	0.005	0.000	0.002
Political Experience	Local MP	0.791	0.322	0.106	0.000	0.010	0.000	0.004	0.012	0.000
Political Experience	National MP 1 Term	0.315	0.186	0.004	0.000	0.430	0.716	0.000	0.134	0.088
Political Experience	National MP 2+ Terms	0.156	0.126	0.000	0.000	0.000	0.306	0.000	0.035	0.018
Educational Attainment	High School	0.005	0.000	0.271	0.618	0.000	0.000	0.024	0.002	0.005
Educational Attainment	College	0.794	0.368	0.000	0.000	0.308	0.858	0.000	0.175	0.008
Educational Attainment	Graduate School	0.404	0.028	0.000	0.000	0.530	0.023	0.000	0.264	0.000
Marital Status	Married	0.007	0.031	0.033	0.000	0.246	0.211	0.000	0.512	0.017
Marital Status	Never Married	0.000	0.980	0.104	0.000	0.000	0.008	0.000	0.000	0.003
Marital Status	Divorced	0.005	0.007	0.007	0.000	0.000	0.001	0.000	0.256	0.000
Youngest Child	No Child	0.024	0.000	0.135	0.068	0.000	0.001	0.004	0.099	0.221
Youngest Child	Younger than 6	0.002	0.350	0.038	0.000	0.040	0.014	0.000	0.036	0.001
Youngest Child	6 to 17	0.035	0.098	0.000	0.000	0.767	0.113	0.020	0.345	0.000
Youngest Child	18 or Older	0.277	0.571	0.002	0.000	0.135	0.346	0.000	0.140	0.006
Residence	Live Far from Parents	0.007	0.015	0.196	0.000	0.000	0.001	0.000	0.168	0.001
Residence	Live Close to Parents	0.720	0.600	0.001	0.000	0.009	0.319	0.000	0.030	0.002
Residence	Live with Parents	0.137	0.168	0.056	0.000	0.204	0.004	0.000	0.061	0.001

p−values from significance test

Candidate's Party: a Ruling party, a Opposition party, a Independent

Note: H0=0.5 for preference and expectation tasks. H0=0 for preference−expecation gap.

Figure A30: p-values from significance tests in full results from Experiment 1, moderated by candidate's party

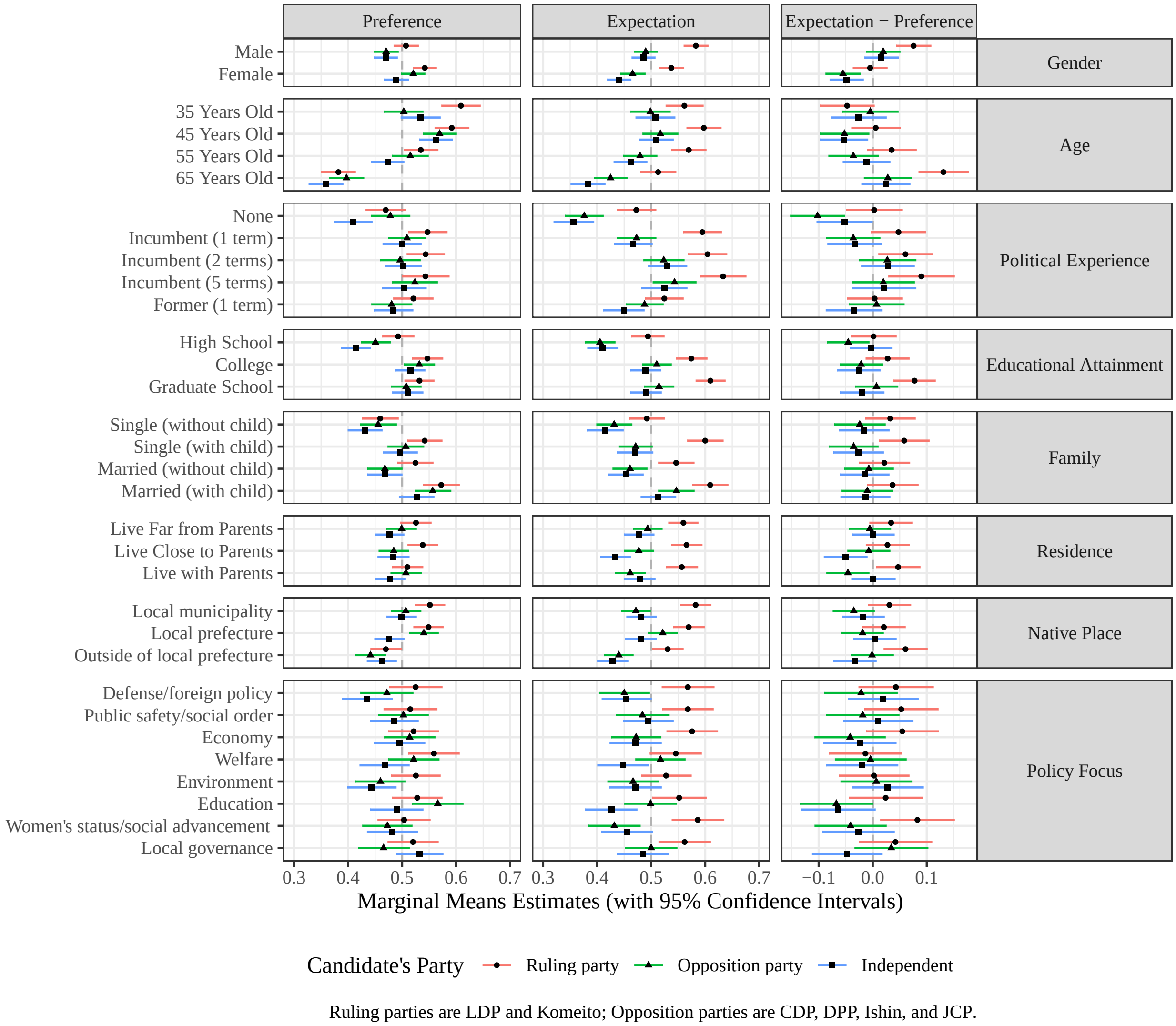

Figure A31: Full results from Experiment 2 (House of Representatives), moderated by candidate's party

		Preference			Expectation			Expectation − Preference		
		Ruling party	Opposition party	Independent	Ruling party	Opposition party	Independent	Ruling party	Opposition party	Independent
Gender	Male	0.543	0.013	0.008	0.000	0.366	0.209	0.000	0.235	0.316
	Female	0.000	0.072	0.338	0.002	0.004	0.000	0.766	0.001	0.003
Age	35 Years Old	0.000	0.873	0.069	0.001	0.938	0.686	0.064	0.867	0.317
	45 Years Old	0.000	0.000	0.000	0.000	0.312	0.591	0.808	0.024	0.018
	55 Years Old	0.033	0.375	0.090	0.000	0.197	0.016	0.131	0.126	0.608
	65 Years Old	0.000	0.000	0.000	0.444	0.000	0.000	0.000	0.217	0.291
Political Experience	None	0.113	0.237	0.000	0.138	0.000	0.000	0.920	0.000	0.046
	Incumbent (1 term)	0.011	0.617	1.000	0.000	0.140	0.064	0.065	0.160	0.196
	Incumbent (2 terms)	0.015	0.844	0.905	0.000	0.224	0.099	0.018	0.317	0.265
	Incumbent (5 terms)	0.054	0.269	0.860	0.000	0.036	0.267	0.004	0.508	0.495
	Former (1 term)	0.272	0.311	0.385	0.177	0.488	0.008	0.894	0.782	0.191
Educational Attainment	High School	0.622	0.000	0.000	0.705	0.000	0.000	0.946	0.023	0.862
	College	0.001	0.028	0.272	0.000	0.464	0.473	0.183	0.287	0.200
	Graduate School	0.023	0.602	0.482	0.000	0.302	0.518	0.000	0.730	0.340
Family	Single (without child)	0.020	0.010	0.000	0.626	0.000	0.000	0.174	0.318	0.495
	Single (with child)	0.011	0.696	0.821	0.000	0.070	0.076	0.013	0.129	0.264
	Married (without child)	0.145	0.056	0.049	0.006	0.018	0.005	0.370	0.758	0.526
	Married (with child)	0.000	0.001	0.111	0.000	0.006	0.429	0.128	0.679	0.565
Residence	Live Far from Parents	0.081	0.947	0.096	0.000	0.640	0.113	0.096	0.783	0.961
	Live Close to Parents	0.008	0.277	0.275	0.000	0.106	0.000	0.182	0.712	0.015
	Live with Parents	0.505	0.630	0.116	0.000	0.006	0.156	0.025	0.024	0.961
Native Place	Local municipality	0.000	0.631	0.950	0.000	0.041	0.193	0.127	0.077	0.385
	Local prefecture	0.001	0.004	0.090	0.000	0.125	0.182	0.314	0.347	0.840
	Outside of local prefecture	0.038	0.000	0.007	0.037	0.000	0.000	0.003	0.951	0.100
Policy Focus	Defense/foreign policy	0.320	0.261	0.006	0.006	0.037	0.051	0.224	0.532	0.567
	Public safety/social order	0.550	0.920	0.525	0.005	0.522	0.837	0.132	0.594	0.769
	Economy	0.378	0.562	0.840	0.002	0.234	0.240	0.108	0.213	0.485
	Welfare	0.015	0.374	0.168	0.064	0.467	0.031	0.697	0.904	0.560
	Environment	0.274	0.089	0.015	0.244	0.166	0.233	0.947	0.845	0.414
	Education	0.244	0.006	0.691	0.042	0.960	0.003	0.493	0.052	0.070
	Women's status/social advancement	0.886	0.248	0.440	0.000	0.005	0.066	0.018	0.229	0.441
	Local governance	0.403	0.158	0.145	0.012	1.000	0.541	0.222	0.322	0.151

p−values from significance test

Respondent's Gender: Ruling party, Opposition party, Independent

Note: H0=0.5 for preference and expectation tasks. H0=0 for preference−expecation gap.

Figure A32: p-values from significance tests in full results from Experiment 2 (House of Representatives), moderated by candidate's party

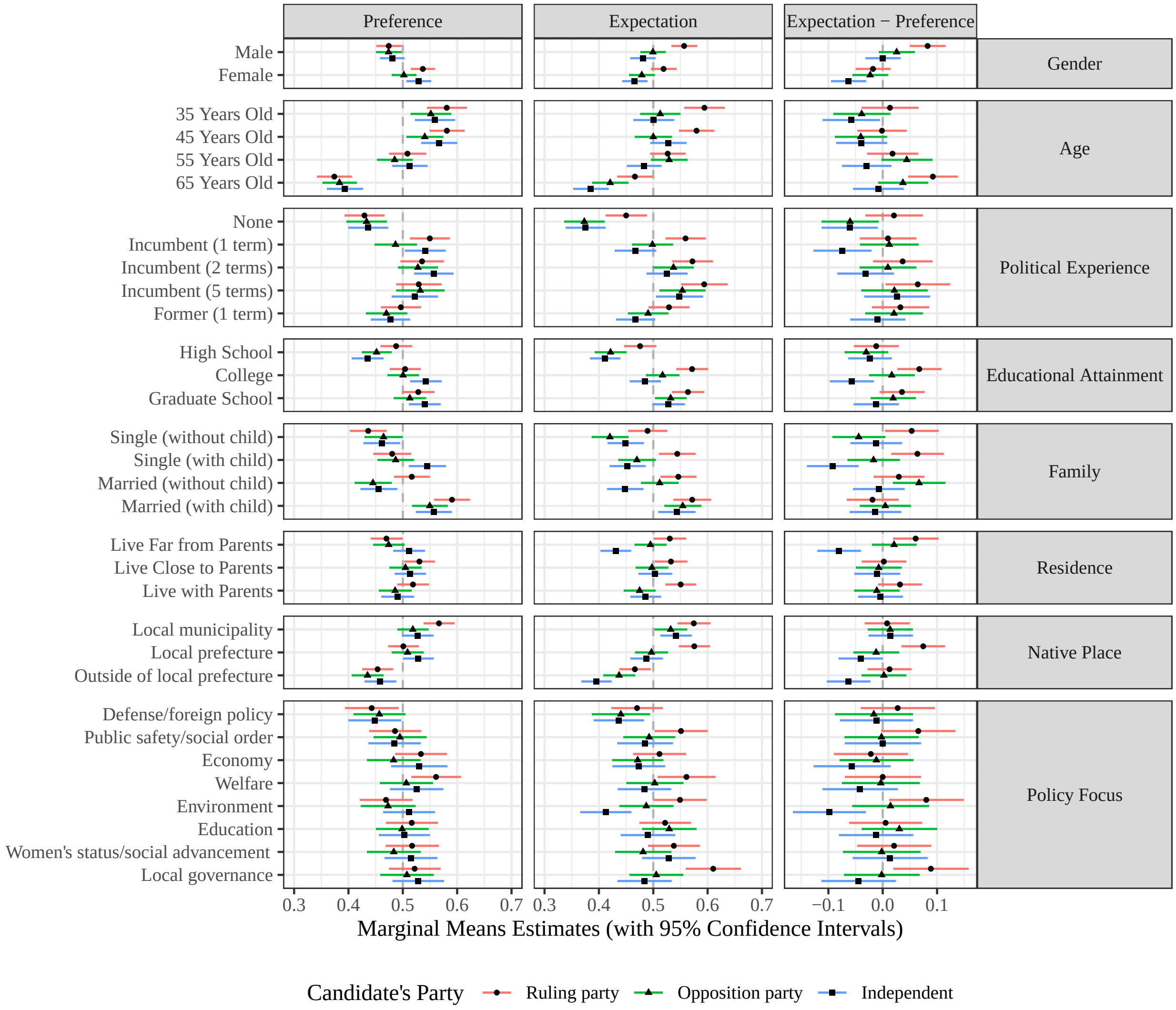

Figure A33: Full results from Experiment 2 (Municipal Council), moderated by candidate's party

Attribute group	Level	Preference: Ruling party	Preference: Opposition party	Preference: Independent	Expectation: Ruling party	Expectation: Opposition party	Expectation: Independent	Expectation − Preference: Ruling party	Expectation − Preference: Opposition party	Expectation − Preference: Independent
Gender	Male	0.026	0.027	0.085	0.000	0.955	0.102	0.000	0.125	0.983
Gender	Female	0.001	0.866	0.011	0.109	0.078	0.003	0.271	0.164	0.000
Age	35 Years Old	0.000	0.006	0.002	0.000	0.496	0.968	0.617	0.145	0.029
Age	45 Years Old	0.000	0.018	0.000	0.000	1.000	0.101	0.951	0.096	0.100
Age	55 Years Old	0.619	0.362	0.435	0.105	0.088	0.294	0.450	0.063	0.196
Age	65 Years Old	0.000	0.000	0.000	0.042	0.000	0.000	0.000	0.109	0.729
Political Experience	None	0.000	0.000	0.001	0.009	0.000	0.000	0.440	0.024	0.020
Political Experience	Incumbent (1 term)	0.007	0.494	0.030	0.002	0.935	0.087	0.713	0.664	0.006
Political Experience	Incumbent (2 terms)	0.080	0.136	0.002	0.000	0.047	0.189	0.185	0.719	0.230
Political Experience	Incumbent (5 terms)	0.165	0.155	0.312	0.000	0.012	0.028	0.032	0.490	0.392
Political Experience	Former (1 term)	0.845	0.116	0.205	0.129	0.615	0.073	0.221	0.442	0.711
Educational Attainment	High School	0.400	0.000	0.000	0.103	0.000	0.000	0.567	0.133	0.243
Educational Attainment	College	0.769	0.974	0.004	0.000	0.270	0.301	0.001	0.435	0.005
Educational Attainment	Graduate School	0.055	0.393	0.006	0.000	0.029	0.064	0.092	0.361	0.561
Family	Single (without child)	0.000	0.046	0.023	0.566	0.000	0.002	0.033	0.072	0.613
Family	Single (with child)	0.265	0.439	0.009	0.009	0.084	0.005	0.009	0.487	0.000
Family	Married (without child)	0.322	0.001	0.009	0.006	0.499	0.002	0.208	0.006	0.757
Family	Married (with child)	0.000	0.003	0.001	0.000	0.002	0.012	0.435	0.845	0.570
Residence	Live Far from Parents	0.041	0.072	0.440	0.044	0.726	0.000	0.004	0.314	0.000
Residence	Live Close to Parents	0.031	0.745	0.347	0.031	0.865	0.825	0.922	0.727	0.631
Residence	Live with Parents	0.203	0.349	0.522	0.000	0.091	0.319	0.121	0.607	0.833
Native Place	Local municipality	0.000	0.202	0.064	0.000	0.034	0.004	0.702	0.514	0.486
Native Place	Local prefecture	0.946	0.559	0.044	0.000	0.822	0.415	0.000	0.570	0.049
Native Place	Outside of local prefecture	0.001	0.000	0.005	0.020	0.000	0.000	0.540	0.920	0.002
Policy Focus	Defense/foreign policy	0.022	0.074	0.035	0.213	0.028	0.007	0.427	0.651	0.730
Policy Focus	Public safety/social order	0.553	0.830	0.530	0.039	0.753	0.560	0.059	0.947	0.997
Policy Focus	Economy	0.167	0.496	0.253	0.643	0.229	0.274	0.526	0.733	0.114
Policy Focus	Welfare	0.008	0.797	0.310	0.023	0.919	0.507	0.999	0.920	0.235
Policy Focus	Environment	0.207	0.287	0.645	0.047	0.610	0.000	0.021	0.691	0.004
Policy Focus	Education	0.495	0.958	0.918	0.366	0.247	0.690	0.877	0.386	0.718
Policy Focus	Women's status/social advancement	0.490	0.505	0.546	0.117	0.476	0.254	0.547	0.956	0.700
Policy Focus	Local governance	0.365	0.764	0.241	0.000	0.827	0.517	0.012	0.955	0.201

p−values from significance test

Respondent's Gender: Ruling party; Opposition party; Independent

Note: H0=0.5 for preference and expectation tasks. H0=0 for preference−expecation gap.

Figure A34: p-values from significance tests in full results from Experiment 2 (Municipal Council), moderated by candidate's party

G Pre-analysis plan for the first experiment: Preference-expectation gap in support for female candidates in Japanese parliamentary elections

Registration Metadata

Title

Preference-expectation gap in support for female candidates in Japanese parliamentary elections

Description

This study examines the extent to which personal preference for female candidates in Japanese parliamentary elections correlates with personal evaluations of female candidates' likelihood of winning.

Contributors

Anonymous contributor(s)

License

MIT License, 2022, Anonymous contributor(s)

Subjects

Social and Behavioral Sciences, Political Science, Comparative Politics

Study Information

Hypotheses

> H1: Individuals are personally not less likely to prefer/view as desirable female political candidates than male political candidates.
>
> H2: Individuals believe other individuals are less likely to prefer/view as desirable female political candidates than male political candidates. i.e. Individuals believe female political candidates are less electable (i.e. likely to win) than male political candidates.

Design Plan

Study type

Experiment - A researcher randomly assigns treatments to study subjects, this includes field or lab experiments. This is also known as an intervention experiment and includes randomized controlled trials.

Blinding

For studies that involve human subjects, they will not know the treatment group to which they have been assigned.

Is there any additional blinding in this study?

N/A

Study design

A conjoint survey experiment whereby respondents review profiles of two hypothetical candidates randomly generated from a set of attributes (see attached document) and then choose between the candidates. All subjects review 6 pairs of profiles. For 3 profiles, subjects choose the candidate they personally prefer/view as desirable. For 3 profiles, they choose the candidate they think a majority of other subjects prefer/view as desirable (i.e. is more likely to win).

We also collect personal information from subjects, including their gender, age, education, marital status, residential environment, annual household income, and partisanship.

Attached are the survey instrument in Japanese, as well as a draft of the English translation.

`experiment_fempoljp_spring22_v2.1.docx`
`experiment_fempoljp_spring21_v2.1_en.txt`

Randomization

To avoid learning effects, we randomize the order in which subjects are asked to make two types of decisions: select the candidate they personally prefer/view as desirable and select the candidate they think a majority of other subjects prefer/view as desirable.

We also randomize attribute levels across conjoint tasks. See section on "Variables" for constraints on how attribute levels are randomized.

In any conjoint task, we maintain the same order of the attributes, as listed in the manipulated variable section. The order is typical of the realistic candidate profile to ensure external validity.

Sampling Plan

Existing Data

Registration prior to creation of data

Explanation of existing data

N/A

Data collection procedures

Participants will be recruited from the monitor pool of the online survey company, Rakuten Insight, with the criteria that they are 18 or older. The entire experiment will be conducted in Japanese.

Sample size

n=1800

Sample size rationale

Since the unit of analysis is rated candidate profile, 1800 subjects who each rate 6 candidate profiles provide us with sufficient power.

Stopping rule

N/A

Variables

Manipulated Variables

Conditions:

A binary measure of whether respondents evaluate candidate profiles in the context of national (House of Representatives) or local election (municipal council).

Conjoint attributes and levels:

1. Gender (性別): Male (男性); Female (女性)
2. Party (所属政党): Liberal Democratic Party/LDP (自民党); Komeito (公明党); Constitutional Democratic Party/CDP (立憲民主党); Democratic Party for the People/DPFP (国民民主党); Japanese Communist Party/JCP (共産党); Japan Ishin Party (日本維新の会); Independent (無所属)
3. Age (年齢): 35 (35歳); 45 (45歳); 55 (55歳); 65 (65歳)
4. Political Experience (政治経験): No Experience (経験なし); Local parliament member (地方議員); One term as a Diet Member (国会議員１期); Two terms or more as a Diet Member (国会議員２期以上)
5. Education (最終学歴): High School (高校卒); College (大学卒); Graduate School (大学院卒)
6. Marital status (結婚歴): Unmarried (未婚); Married (既婚); Divorced (離別)
7. Children Status (子の年齢 (一番下)): No Children (子なし); Children of 6- (６歳未満); Children of 6+ (６－１7歳); Children of 18+ (１８歳以上)

8. Residential Status (居住状況) : Live together with parent (親と共に居住); Live close to parent (親の近くに居住); Live far from parent (親から遠くに居住)

Constraints:

Parties are constrained so that a candidate from ruling party (LDP or Komeito) is always matched with one from opposition (All else).

Measured variables

Outcome variables:

1. Perception of which candidate is more desirable/preferred
2. Perception of which candidate is more likely to win

Covariates include: [see attached file for measurement]:

1. Gender and Age [genderage]
2. Education [edu]
3. Residential environment [urban], [postcode]
4. Socioeconomic status: income [income], occupation [employment]
5. Family status: married and have kids or not [marrykids]
6. Political views: support for the current Kishida cabinet [pmsup], interest in politics [polint], vote in the 2021 election [voted], partisanship [psup]
7. Political knowledge: Self-assessed levels of political knowledge [selfknow] and aggregated score of answers to 9 factual test questions. Following Delli Carpini and Keeter (1996), three questions are on political institutions, four questions are on ideological position of parties (LDP versus CDP, LDP versus JCP, Ishin versus CDP, Ishin versus JCP), and two questions are on political leaders. We will use both the continuous scale and the binary scale (median split). [know_court, know_cabinet; know_hoc; ppos_2; ppos_3; ppos_5; ppos_6; know_kishi; know_makishima]

Indices

To create a continuous scale of aggregated variables, we sum up question responses and rescale to a unit interval.

To create a binary scale of aggregated variables, we sum up question responses and recode those above the midpoint as 1, those below the midpoint as 0.

Analysis Plan

Statistical models

We estimate the average marginal component effect (AMCE) of each attribute on the probability that the candidate will be chosen as the preferred candidate as well as the more likely to win candidate, where the average is taken over all possible combinations of the other candidate attributes. We run the analysis separately for each dependent variable and for national/local election conditions.

Transformations

Continuous variables will be standardized in the standard deviation units.

Inference criteria

Alpha = .05 and 0.10; two-tailed.

Data exclusion

Cases who take too long or too short time (3SD above/below the mean) to complete each conjoint task are excluded.

Missing data

If respondents do not complete all conjoint tasks, we retain the choices they did make.

Exploratory analysis

We expect certain candidate attributes moderate the relationship between their gender and level of support. Therefore, we will look for interaction effects between gender and political party, education, marital status, children status, and residential status.

We expect certain voter attributes moderate the relationship between candidate gender and level of support. Therefore, we will look for interaction effects between candidates' gender and voters' gender, political party, marital status, children status, education residential environment.

Other

Other

N/A

H Pre-analysis plan for the second experiment: Preference-expectation gap in support for female candidates in Japanese national and local elections

Registration Metadata

Title

Preference-expectation gap in support for female candidates in Japanese national and local elections

Description

This study examines the extent to which personal preference for female candidates in Japanese parliamentary elections correlates with personal evaluations of female candidates' likelihood of winning. Furthermore, it tests whether this correlation varies by national or local-level election.

Contributors

Anonymous contributor(s)

License

MIT License, 2022, Anonymous contributor(s)

Subjects

Social and Behavioral Sciences, Political Science, Comparative Politics

Study Information

Hypotheses

> H1: Individuals are personally not less likely to prefer/view as desirable female political candidates than male political candidates.
>
> H2: Individuals believe other individuals are less likely to prefer/view as desirable female political candidates than male political candidates. In other words, individuals believe female political candidates are less electable (i.e. less likely to win) than male political candidates.
>
> H3: The preference-electability gap is wider in national elections than in local elections. In both national and local elections, individuals are personally not less likely to prefer/view as desirable female political candidates than male political candidates. More so in national than local elections, individuals believe other individuals are less likely to prefer/view as desirable female political candidates than male political candidates.

Design Plan

Study type

Experiment - A researcher randomly assigns treatments to study subjects, this includes field or lab experiments. This is also known as an intervention experiment and includes randomized controlled trials.

Blinding

For studies that involve human subjects, they will not know the treatment group to which they have been assigned.

Is there any additional blinding in this study?

N/A

Study design

Respondents are randomized into two conditions: national election (House of Representatives) and local election (municipal council). Within each condition, a conjoint survey experiment whereby respondents review profiles of two hypothetical candidates randomly generated from sets of attributes and then choose between the candidates. All subjects review 8 pairs of profiles. For 4 profiles, subjects choose the candidate they personally prefer/view as desirable. For 4 profiles, they choose the candidate they think is more likely to get elected (i.e. a majority of other subjects prefer/view as desirable).

The specific attributes that are randomized are described below. Attribute order is randomized across each respondent (gender -¿ age and family structure -¿ living condition orders are preserved to make a profile look natural).

We also collect personal information from subjects, including their gender, age, education, marital status, residential environment, annual household income, and partisanship.

Attached are the entire survey instrument in Japanese, as well as a draft of the English translation.

```
experiment_fempoljp_spring22_v3.1.docx
experiment_fempoljp_spring21_v3.1_en.txt
```

Randomization

To avoid learning effects, we randomize the order in which subjects are asked to make two types of decisions: select the candidate they personally prefer/view as desirable and select the candidate they think is more likely to be elected.

We also randomize attribute levels across conjoint tasks and also attribute orders across respondents. See section on ”Variables” for constraints on how attribute levels are randomized.

In any conjoint task within the same respondent, we maintain the same order of the attributes, but the order is randomized across respondents to avoid ordering effects.

Sampling Plan

Existing Data

Registration prior to creation of data

Explanation of existing data

N/A

Data collection procedures

Participants will be recruited from the monitor pool of the online survey company, Rakuten Insight, with the criteria that they are 18 or older. The entire experiment will be conducted in Japanese.

Sample size

n=2400

Sample size rationale

According to Orme 2019 (https://sawtoothsoftware.com/resources/technical-papers/sample-size-issues-for-conjoint-analysis-studies), the minimum required N for conjoint experiment is calculated by $(n*t*a)/c \geq 500$ where n is the number of respondents, t is the number of tasks, a is number of alternatives per task (not including the none alternative), and c is equal to the largest number of levels for any one attribute (p.64). In our experiment, n is 1200 (under each election context), t is 4, a is 2, and c is 8 (see Variable section) and thus $(n*t*a)/c = (1200*4*2)/8 = 1200 > 500$, and satisfying this minimal sample size.

Stopping rule

N/A

Variables

Manipulated Variables

Conditions:

A binary measure of whether respondents evaluate candidate profiles in the context of national (House of Representatives) or local election (municipal council).

Conjoint attributes and levels:

1. party affiliation: ruling party in the Diet; opposition party in the Diet; No affiliation/minor party (所属政党: 国会における与党、国会における野党、無所属・諸派)
2. gender: male; female (性別：男性、女性)

3. age: 35; 45; 55; 65 (年齢)
4. educational attainment: high school, university, graduate school (最終学歴：高校卒、大学卒、大学院卒)
5. political experience: no experience; incumbent (1 term); incumbent (2 terms); incumbent (5 terms); former (1 term) (政治経験：経験なし、現職（１期）、現職（２期）、現職（５期）、元職（１期）)
6. family structure: single & have children, single & no children, married & have children, married & no children (家族構成：独身で子がいる、独身で子はいない、結婚して子がいる、結婚して子はいない)
7. living condition: live with parents, live close to parents, live far from parents (居住状況：親と共に居住、親の近くに居住、親から遠くに居住)
8. policy focus: defense/foreign policy; safety/social order; economy; welfare; environment; education; women's status and social advancement; local governance (重点政策分野：防衛・外交、治安・社会秩序、経済、福祉、環境、教育、女性の地位・社会進出、地方自治)
9. area of origin: local municipality, local prefecture, non-local prefecture (出身地：地元の市区町村、地元の都道府県、地元ではない都道府県)

Constraints:

Attribute order sets, gender to age, and family structure to living conditions are always preserved to make the profile look natural.

Measured variables

Outcome variables:

1. Perception of which candidate is more desirable/preferred
2. Perception of which candidate is more likely to win

Covariates include: [see attached file for measurement]:

1. Gender and Age [genderage]
2. Education [edu]
3. Residential environment [urban], [postcode]
4. Socioeconomic status: income [income], occupation [employment]
5. Family status: married and have kids or not [marrykids]
6. Political views: support for the current Kishida cabinet [pmsup], interest in politics [polint], vote in the 2021 election [voted], partisanship [psup]

7. Political knowledge: Self-assessed levels of political knowledge [selfknow] and aggregated score of answers to 9 factual test questions. Following Delli Carpini and Keeter (1996), three questions are on political institutions, four questions are on ideological position of parties (LDP versus CDP, LDP versus JCP, Ishin versus CDP, Ishin versus JCP), and two questions are on political leaders. We will use both the continuous scale and the binary scale (median split). [know_court, know_cabinet; know_hoc; ppos_2; ppos_3; ppos_5; ppos_6; know_kishi; know_makishima]

Indices

To create a continuous scale of aggregated variables, we sum up question responses and rescale to a unit interval.

To create a binary scale of aggregated variables, we sum up question responses and recode those above the midpoint as 1, those below the midpoint as 0.

Analysis Plan

Statistical models

We estimate the average marginal component effect (AMCE) of each attribute on the probability that the candidate will be chosen as the preferred candidate as well as the more likely to win candidate, where the average is taken over all possible combinations of the other candidate attributes. We run the analysis separately for each dependent variable and for national/local election conditions.

Transformations

Continuous variables will be standardized in the standard deviation units.

Inference criteria

Alpha = .05 and 0.10; two-tailed.

Data exclusion

Cases who take too long or too short time (3SD above/below the mean) to complete each conjoint task are excluded.

Missing data

If respondents do not complete all conjoint tasks, we retain the choices they did make.

Exploratory analysis

We expect certain candidate attributes to moderate the relationship between their gender and level of support. Therefore, we will look for interaction effects between gender and age, political party, political experience, education, family structure, residential status, policy focus, and the area of origin.

We expect certain voter attributes to moderate the relationship between candidate gender and level of support. Therefore, we will look for interaction effects between candidates' gender and voters' gender, age, political party, family structure, education, residential environment, political knowledge and policy focus.

Other

Other

N/A
